# Single-cell transcriptome reveals dominant subgenome expression and transcriptional response to heat stress in Chinese cabbage

**DOI:** 10.1186/s13059-022-02834-4

**Published:** 2022-12-19

**Authors:** Xiaoxue Sun, Daling Feng, Mengyang Liu, Ruixin Qin, Yan Li, Yin Lu, Xiaomeng Zhang, Yanhua Wang, Shuxing Shen, Wei Ma, Jianjun Zhao

**Affiliations:** State Key Laboratory of North China Crop Improvement and Regulation, Key Laboratory of Vegetable Germplasm Innovation and Utilization of Hebei, Collaborative Innovation Center of Vegetable Industry in Hebei, College of Horticulture, Hebei Agricultural University, Baoding, 071000 China

**Keywords:** Chinese cabbage, Single-cell RNA sequencing, Whole-genome triplication, Subgenome dominance, Leaf development, Heat stress

## Abstract

**Background:**

Chinese cabbage (Brassica rapa ssp. pekinensis) experienced a whole-genome triplication event and thus has three subgenomes: least fractioned, medium fractioned, and most fractioned subgenome. Environmental changes affect leaf development, which in turn influence the yield. To improve the yield and resistance to different climate scenarios, a comprehensive understanding of leaf development is required including insights into the full diversity of cell types and transcriptional networks underlying their specificity.

**Results:**

Here, we generate the transcriptional landscape of Chinese cabbage leaf at single-cell resolution by performing single-cell RNA sequencing of 30,000 individual cells. We characterize seven major cell types with 19 transcriptionally distinct cell clusters based on the expression of the reported marker genes. We find that genes in the least fractioned subgenome are predominantly expressed compared with those in the medium and most fractioned subgenomes in different cell types. Moreover, we generate a single-cell transcriptional map of leaves in response to high temperature. We find that heat stress not only affects gene expression in a cell type-specific manner but also impacts subgenome dominance.

**Conclusions:**

Our study highlights the transcriptional networks in different cell types and provides a better understanding of transcriptional regulation during leaf development and transcriptional response to heat stress in Chinese cabbage.

**Supplementary Information:**

The online version contains supplementary material available at 10.1186/s13059-022-02834-4.

## Background

Chinese cabbage (*Brassica rapa* ssp. *pekinensis*) is an economically important vegetable crop that is cultivated worldwide. The main agriculturally important organ of Chinese cabbage is the leafy head, which results from leaf curvature and is directly responsible for yield and marketability. Thus, leaf traits are often the primary targets for breeders. Leaf develops initially from shoot apical meristem and undergoes founder cell recruitment, distal growth, blade initiation, and intercalary growth to reach its final shape [1]. Leaf development is controlled by genes that are involved in multiple physiological pathways, along with coordinated cell patterning [2]. Chinese cabbage has undergone a whole-genome triplication (WGT) event during its evolution and domestication. The genome of Chinese cabbage has evolved to comprise three subgenomes, namely, the least, medium, and most fractionated subgenomes (LF, MF1, and MF2, respectively). Such WGT and subgenome dominance along with biased gene retention have also propelled the expansion of diverse morphotypes and increased the total gene number by approximately three folds in Chinese cabbage when compared to *Arabidopsis* [3]. This makes genetic and genomic analysis of diploid Chinese cabbage (A genome, *n* = 10) even more challenging. On the other hand, cells are basic building blocks for life and regulatory units to modulate leaf architecture. Therefore, to fundamentally understand leaf development, it needs to analyze physiological, genetic, and molecular processes within individual cells [4]. Single-cell RNA sequencing (scRNA-seq) technology provides an unprecedented opportunity to generate large quantities of data for studying tissue/organ development at single-cell resolution [5]. Despite extensive studies, there is no comprehensive analysis of identities of cell types in the leaves of *Brassica* plants [6, 7]. Moreover, the underlying mechanisms of triplicated gene retention and the functional divergence of triplicated genes remain unclear. The use of scRNA-seq can help understanding expression dominance and provide new insights into gene dose balance within the subgenome at the cellular level in Chinese cabbage.

Global climate change leads to extreme temperatures, which are major abiotic stresses that limit the growth and production of plants [8]. Leaves serve as an interface between plants and the environment and respond to environmental stimuli. For instance, after exposure to high temperature, the leaf photosynthetic rate decreases, influencing plant productivity [9]. Especially for Chinese cabbage, a cool-season leafy vegetable species, high temperature affects the quantity and quality of leafy head formation and results in low harvestable yields [10]. The main defensive response to heat stress involves heat shock proteins (HSPs) that functionally target heat stress responsive transcription factors (TFs) to control heat stress-inducible gene expression [11]. Previous studies have revealed that cells tightly regulate gene expression by altering the transcriptional capacity under heat stress. However, knowledge about transcriptional regulatory network underlying the heat stress response at the cellular level is currently lacking [12]. Recent genome-wide analyses of transcription with scRNA-seq data provide a new picture of the molecular basis of gene expression regulation for different cell types in plant response to stress.

Here, we isolated protoplasts from the shoots and leaves of Chinese cabbage seedlings for scRNA-seq. A transcriptional map of the Chinese cabbage leaves at a single-cell resolution was subsequently generated. We classified six major cell types and identified several potential cell-type-specific marker genes in these heterogeneous cell populations. Using the *B. rapa* genome as a model system, we investigated the role of WGT in speciation and morphotype diversification. The subgenome dominance effect and biased gene retention were analyzed at the single-cell level in Chinese cabbage. Furthermore, we performed scRNA-seq on Chinese cabbage leaf cells under heat stress conditions. Through mining the single-cell transcriptome data, we identified cell-type-specific regulation of gene expression and detected changes in gene expression patterns and expression variations of multiple-copy genes in different cell populations under heat stress. Overall, we reported novel data about mRNA transcripts involved in leaf development, subgenome dominance effects, and transcriptional responses to heat stress at the cellular level. Our findings will enable improvements in Chinese cabbage cultivation and promote plant tolerance to heat stress for agricultural applications.

## Results

### Single-cell RNA sequencing of Chinese cabbage shoot and leaf cells

**Fig. 1:**
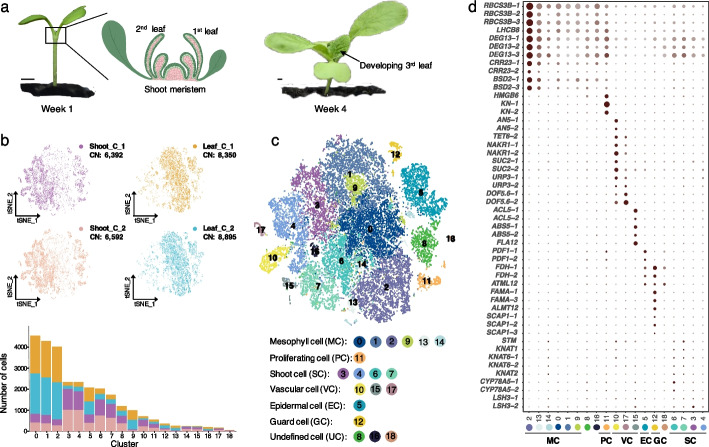
Cell heterogeneity within Chinese cabbage shoots and leaves. **a** Chinese cabbage shoot apices and developing leaves were used for protoplast isolation. **b** Distribution and numbers of cells for two biological replicates of shoot and leaf samples. **c** t-SNE visualization for the identification of 19 cell clusters from 28,343 cells in shoots and leaves. **d** Expression pattern of representative cell-type marker genes in 19 cell clusters. The average expression level (color) and the proportion of cells expressing the gene (dot size) are shown

To systematically determine gene expression patterns during Chinese cabbage leaf development, we isolated protoplasts from inbreeding line Chinese cabbage A03 shoot apices (0.5 cm in length from the shoot tip) (S) and developing leaves (L) and profiled them using droplet-based scRNA-seq to generate a single-cell transcriptomic atlas (Fig. 1a). After enzymatic digestion, shoot and leaf cells were isolated from 1-week-old and 4-week-old plants grown at 25 °C day/18 °C night, respectively. To monitor the reproducibility of the experiment and reliability of the scRNA-seq results, two replicates were included for both shoot and leaf cell samples. A total of 12,985 individual shoot cells (6392 in S1 and 6592 in S2) and 17,245 individual leaf cells (8350 in L1 and 8895 in L2) were labeled (Fig. 1b). Then, cDNA libraries were generated and sequenced, and the data were filtered at both the cell and gene levels. Approximately 64,000 reads and 1600 median genes per shoot cell and 51,000 reads and 3400 median genes per leaf cell were detected for further analysis (Additional file 2: Table S1).

To generate a cell atlas of Chinese cabbage leaf development, we merged two shoot apex samples (S1 and S2) with two leaf samples (L1 and L2) for cell clustering and annotation. In total, 28,343 single-cell transcriptomes were used to identify distinct cell populations, and they were grouped into 19 distinct clusters (Additional file 3: Table S2; Additional file 1: Figure S1). The t-distributed stochastic neighborhood embedding (t-SNE) method was used to visualize and explore the cell clusters (Fig. 1c). These clusters harbored similar numbers of cells in each replicate but showed differences between shoot and leaf samples (S/L) in terms of the proportion of these two cell types. Clusters 3, 4, 6, 7, 8, 10, 11, 14, 15, 17, and 18 contained significantly more cells from shoot samples (S), while clusters 0, 1, 2, 5, 9, 13, and 16 contained significantly more cells from leaf samples (L) (Additional file 1: Figure S1).

### Identification of major cell types in the shoots and leaves

To annotate each cluster, we first identified cluster-enriched genes that were highly expressed in one cluster compared to all the other clusters (the genes must be expressed in 25% of cells within the cluster; *q* ≤ 0.01; log_2_|fold change (FC)| ≥ 0.36) (Additional file 4: Table S3). To explore the potential regulators of different clusters, Gene Ontology (GO) and Kyoto Encyclopedia of Genes and Genomes (KEGG) analyses were performed (Additional file 5: Table S4). The distribution of cluster-enriched genes ranged from 387 to 2021 per cluster, and GO analysis of these gene sets revealed the potential biological functions of the genes expressed in each cell cluster, which helped us to predict cell types. In addition, the expression of a series of marker genes, including those whose functions have been thoroughly studied or those identified from transcriptomic datasets, was compared across clusters to determine the cell types in those clusters (Additional file 1: Figure S2). Some of these marker genes were highly and specifically enriched in each corresponding cluster and helped us to identify major cell types present in the shoots and leaves, which included meristem cells (four clusters), mesophyll cells (six clusters), proliferating cells (one cluster), epidermal cells and guard cells (two clusters), and vascular cells (three clusters) (Additional file 6: Table S5).

The expression of key meristem development genes, such as *SHOOT MERISTEMLESS* (*STM*), KNOTTED 1-LIKE HOMEOBOXs *KNAT1*, *KNAT2* and *KNAT6*, *CYTOCHROME P450* (*CYP78A5*), and *LIGHT SENSITIVE HYPOCOTYLS 3* (*LSH3*), showed high specificity in clusters 3, 4, 6, and 7, which were shoot meristematic cells [13–15]. The proliferating cell marker genes, including *3XHIGH MOBILITY GROUP-BOX 2* (*3xHMG-box2*) and *SYNTAXIN OF PLANTS 111* (*SYP111*), were expressed in cluster 11 cells [16, 17]. The vascular cells were assigned to clusters 10, 15, and 17, in which the following genes were expressed: the companion cell genes *ARATHNICTABA 5* (*AN5*), *SUCROSE-PROTON SYMPORTER 2* (*SUC2*), *TETRASPANIN 6* (*TET6*), and *HEAVY METAL-ASSOCIATED PROTEIN 42* (*NAKR1*); the phloem-related genes *UAS-TAGGED ROOT PATTERNING 3* (*URP3*) and *DNA BINDING WITH ONE FINGER 5.6* (*DOF5.6*); and the xylem-related genes *ACAULIS 5* (*ACL5*), *ABNORMAL SHOOT 5* (*ABS5*), and *FASCICLIN-LIKE ARABINOGALACTAN-PROTEIN 12* (*FLA12*) [6, 18–21]. Clusters 5 and 12 comprise epidermal cells and guard cells. The epidermal cell and guard cell marker genes included *PROTODERMAL FACTOR 1* (*PDF1*), *FIDDLEHEAD* (*FDH*), *MERISTEM LAYER 1* (*ATML1*), *FAMA* (*FMA*), *ALUMINUM-ACTIVATED 12* (*ALMT12*), and *STOMATAL CARPENTER 1* (*SCAP1*) [22–27]. Mesophyll cells were assigned to clusters 0, 1, 2, 9, 13, and 14. The light-dependent gene *RUBISCO SMALL SUBUNIT 3B* (*RBCS3B*) and chloroplast-related genes *CHLORORESPIRATORY REDUCTION 23* (*CRR23*) and *BUNDLE SHEATH DEFECTIVE 2* (*BSD2*) were highly expressed mainly in clusters 2 and 13 and weakly expressed in clusters 0, 1, and 9. These genes encode chloroplast proteins that are highly expressed in the mesophyll cells [6, 7, 28]. In addition, clusters 0 and 2 were highly enriched for the expression of “chloroplast” signature genes, and clusters 2, 13, and 14 were enriched in genes involved in the “photosynthesis” pathway, suggesting that these mesophyll cell populations play pivotal roles in light capture (Additional file 1: Figure S1). Similarly, correlations between clusters could reveal the cell type organization. We found a strong correlation between clusters 0, 1, and 9 (>0.95). Clusters 16 and 18 showed weak correlations (0.2 and 0.1, respectively) with all the other clusters. Using the known marker genes, we could not determine the cell types for clusters 8, 16, or 18.

### Identification of novel cell-type marker genes

Several databases are available for selecting cell types in a few plant species, including *Arabidopsis*, rice, maize, and peanut, but no vegetable species are represented. However, we found that these marker genes are not expressed exclusively in a single cell type, making it desirable to identify novel genes with cell-type-specific expression in Chinese cabbage.

To explore the potential marker genes for different cell types, we analyzed gene expression profiles in shoot meristematic cells, mesophyll cells, epidermal cells, guard cells, vascular cells, and proliferating cells and in three unknown cell clusters, UK8, UK16, and UK18. We confirmed the genes with high (average expression value in the target cluster > that of the others) and cell-type-specific expression (genes must be expressed in 25% of cells within the target cell type and < 25% of cells in all the other cell types; *p* value ≤ 0.01; log_2_FC ≥ 0.5). In total, 24 genes in SCs, 229 genes in mesophyll cells, 78 genes in epidermal cells, 116 genes in guard cells, 72 genes in vascular cells, and 219 genes in proliferating cells as well as 22 genes in UK8, 237 genes in UK16, and 808 genes in UK18 were identified (Additional file 7: Table S6). Some genes with known gene functions were included (Additional file 1: Figure S2): the stomatal guard cell differentiation promote the gene *FAMA* (*BAA01g31960*, *BAA03g42630*) and the putative Na+/H+ antiporter gene *CHX20* (*BAA04g05930*) in guard cells; the plant-specific transmembrane domain-containing protein-encoding gene *MLO6* (*BAA01g31040*) and the wax biosynthesis gene *KCS3* (*BAA09g69430*) in epidermal cells; the cell expansion gene *ARL* (*BAA03g24360*) in shoot meristematic cells; the mitotic cell cycle and division control gene *SCL28* (*BAA09g16200*) in proliferating cells; the large subunit of ADP-glucose pyrophosphorylase-encoding gene *ADG2* (*BAA10g21220*) in mesophyll cells; and the phloem development gene *APL* (*BAA07g26030*, *BAA02g27290*) and the Cu-chaperone protein-encoding gene *CCH* (*BAA07g21830*) in vascular cells. Moreover, many genes whose functions were unknown were identified from the transcriptomic datasets. The top 20 marker genes with the highest expression in each cell type were selected for display in the t-SNE plots to show the cell type specificity (Additional file 1: Figure S2).

### Predominant gene expression from different subgenomes in different cell types

Compared with the model plant species *Arabidopsis*, *Brassica* species experienced a WGT event, which has played an important role in the morphotypic diversification of *Brassica* plants. The genome of Chinese cabbage comprises three subgenomes, namely, LF, MF1 and MF2, which differ in both gene density and gene expression.

**Fig. 2:**
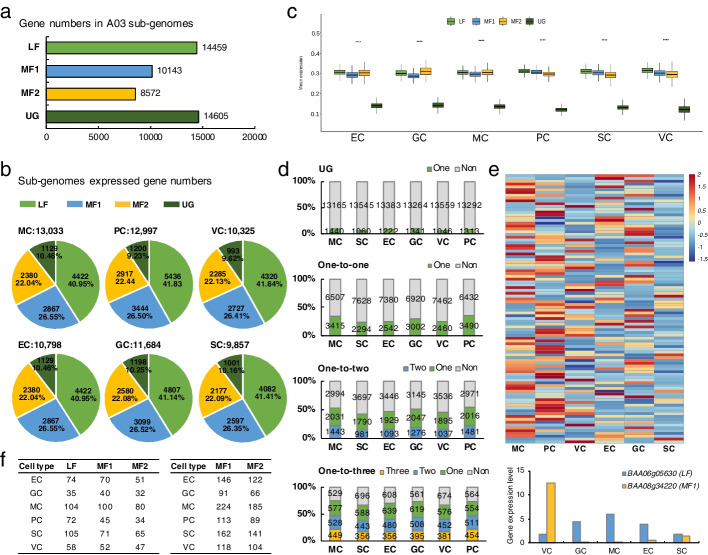
Gene dominance of the A03 subgenomes. **a** Gene number in the A03 subgenomes. **b** Proportion of expressed genes from the subgenomes in different cell types. **c** Mean expression values in the LF, MF1, MF2, and UG in different cell types. **d** Percentage of expressed genes in different cell types for one-to-one-, one-to-two-, and one-to-three-copy genes in A03. The numbers in the figure are in pairs. **e** Gene expression profiles for one-to-two syntenic gene copies expressed in different cell types. **f** Predominance of gene expression in different cell types across the three subgenomes

The Chinese cabbage A03 line was found to contain 14,470 genes (covering 31% of the A03 genome) in LF, 10,160 genes in MF1 (covering 21%), and 8578 genes in MF2 (covering 18%), as well as 14,605 ungrouped genes (UG) (covering 30%) [29] (Fig. 2a). To characterize the genes that shows predominant expression from a specific subgenome, gene expression was measured for all genes in the populations of mesophyll cells, mesophyll cells, epidermal cells, guard cells, vascular cells, and proliferating cells respectively, and expressed genes were identified as those expressed in at least 5% of cells in the target cell type. In total, 13,011 genes in mesophyll cells, 12,997 genes in proliferating cells, 10,325 genes in vascular cells, 10,798 genes in epidermal cells, 11,684 genes in guard cells, and 9857 genes in SCs were identified as expressed genes. In each cell type, the proportion of expressed genes in the subgenome was similar. The expression percentage/density of the subgenomes was in the order of LF>MF1>MF2>UG; 41% showed expression in the LF subgenome in each cell population, 26% showed expression in MF1, 22% showed expression in MF2, and 10% showed expression in the UG (Fig. 2b). The mean expression values of all expressed genes in LF, MF1, and MF2 were similar across different cell types and significantly higher than those in the UG, which contained the largest number of genes (Fig. 2c). Unlike the *B. rapa* genes in the three subgenomes, those in the UG were identified as having no syntenic orthologs in *Arabidopsis.*

The syntenic orthologs between *Arabidopsis* and *Brassica* were identified using the method described by Cheng et al. (2012), and 9922 one-to-one copies, 6468 pairs one-to-two copies, and 2083 groups one-to-three copies of *Brassica* genes were identified in A03. Together with nonsyntenic genes in the UG, we quantified the number of expressed genes from different subgenomes in all six cell types. The UG had the highest number of genes, but the proportion of genes expressed within the group was the lowest—approximately 10%. For the syntenic genes, the proportion of expressed genes within groups was high for one-to-three-copy genes, followed by one-to-two-copy and one-to-one-copy genes (Fig. 2d).

Does a subgenome exhibit dominant expression at the cellular level? An analysis of the transcript levels of genes in different cell types was performed to identify the expression differences among duplicated co-orthologs. We used a twofold change method to evaluate the predominantly expressed genes, or those that were more highly expressed in one subgenome than in the other two subgenomes according to the criteria that at least 5% of cells were expressed in the target cell type and the |log2FC| was ≥0.36 with a *p* value of ≤0.05 (Additional file 8: Table S7). We found that the genes in the LF subgenome were predominantly expressed compared with the genes in MF1 and MF2, especially in SCs and proliferating cells (Fig. 2f). In addition, more genes in MF1 than in MF2 were predominantly expressed. Not only the difference in the expression level but also the function of the predominantly expressed genes in each subgenome differed across different cell types (Additional file 1: Figure S3). For example, in the mesophyll cells, the LF predominantly expressed genes were enriched in phosphatase regulator activity, organonitrogen compound biosynthetic process, and ribosome-related terms; MF1 predominantly expressed genes were enriched in cellular respiration, translation, and ATPase complex transport; and MF2 predominantly expressed genes were enriched in peptide biosynthetic and metabolic processes, intercellular parts, and organelle membrane systems. Although the expression distribution across the three subgenomes was similar in different cell types, the expression patterns of duplicated genes differed between cell types. An analysis of the transcript levels of genes in different cell types revealed the expression differences between duplicated co-orthologs (Fig. 2e). Interestingly, orthologs of *Arabidopsis* genes were predominantly expressed in different cell types. For example, *BAA06g05630* (LF) and *BAA08g34220* (MF1) are homologous to *AT1G08050*, which encodes a zinc finger (C3HC4-type RING finger) protein, and these genes were expressed in epidermal cells and vascular cells, respectively, which may indicate that the copies of these genes had different functions in different cell types.

### Heat stress induced transcriptomic changes vary among cell types

Chinese cabbage is a cool-season leafy vegetable species whose leaf development is greatly influenced by temperature. To study the cellular heterogeneity of Chinese cabbage leaves in response to heat stress, we isolated protoplasts from the third true leaves of 4-week-old plants grown at 40 °C for 12 h (“heat”). Protoplasts from plants grown at 25 °C day/18 °C night were used as controls.

**Fig. 3:**
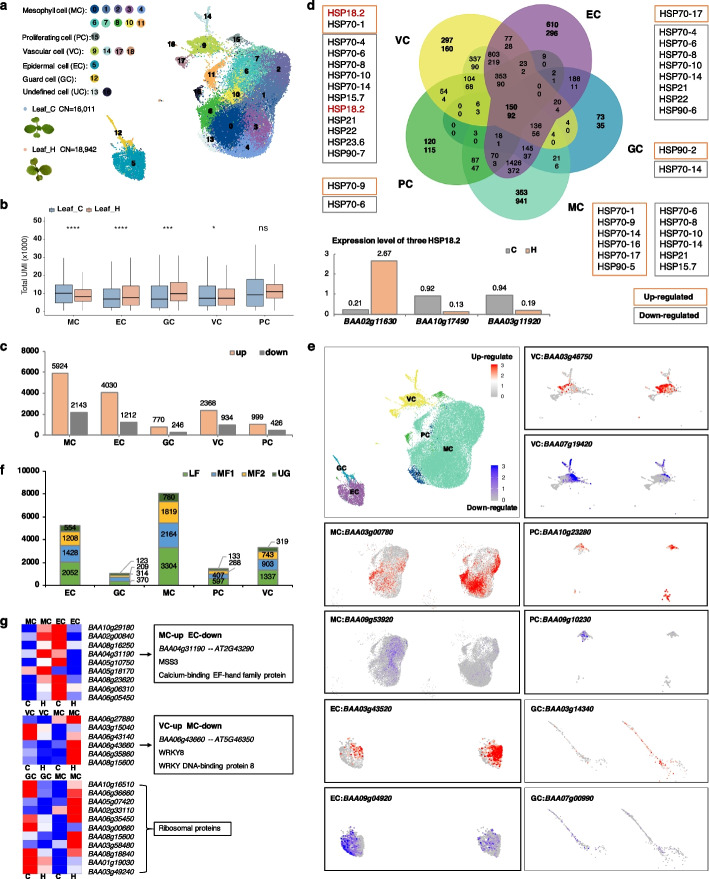
Genes with differential expression patterns under heat stress in different cell types. **a** UMAP plot showing the cell distribution of leaf samples under control and heat stress conditions. **b** Total UMI values for each cell type in control and heat stress leaf samples. **c** DEGs between the control and heat stress treatments in the mesophyll cell, EC, guard cell, vascular cell, and proliferating cell samples. **d** Venn diagram showing overlapping DEGs between different cell types and the distribution of HSP family genes. **e** Heat stress marker genes for different cell types. **f** Numbers of DEGs in the LF, MF1, MF2, and UG subgenomes in different cell types after heat stress. **g** Examples of DEGs with opposite expression patterns in different cell types

In total, 17,245 “control” individual leaf cells (8350 in L1-C and 8895 in L2-C) and 20,663 “heat-treated” individual leaf cells (11,075 in L1-H and 9588 in L2-H) were obtained (Additional file 1: Figure S4). The medians of the unique molecular identifiers (UMIs) and genes were 9618 and 3208 per cell in LC and 8310 and 3546 per cell in LH, respectively. We classified 34,953 leaf cells into 19 clusters, and the uniform manifold approximation and projection (UMAP) algorithm was used to visualize and explore the datasets. All 19 cell clusters included cells from both control and heat-treated plants, which suggests that the cell type identities were not affected by heat treatment (Fig. 3a). However, the percentage of cells in each cluster was notably different between LC and LH (Additional file 1: Figure S4). The aforementioned marker genes were used to determine the cell types of each cluster. In the central part of the leaves, mesophyll cells were found in the most cell clusters, including clusters 0, 1, 2, 3, 4, 6, 7, 8, 10, and 11. Cluster 15 comprised proliferating cells. Phloem cells, companion cells, and xylem cells contained in the vasculature were identified in clusters 9, 14, 17, and 18. Cluster 5 and neighboring cluster 12 comprised epidermal cells and guard cells. Consequently, the major cell types in the leaves were identified, including mesophyll cells, vascular cells, and epidermal cells.

The UMI reflects the number of transcripts captured by scRNA-seq. After heat treatment, we found that the total UMI significantly decreased in the mesophyll cell, epidermal cell, guard cell, and vascular cell types but not in the proliferating cells (Fig. 3b). We measured the expression of genes affected by heat stress in different cell types. The reference genes UBC10, TUA, TSB, CAC, SNF, SAND, UBC1, PP2A, and ZNF were not differentially expressed in different cell types between the control and heat stress treatment groups [30, 31] (Additional file 9: Table S8). Several groups have reported that gene expression patterns were affected after heat treatment in different cell types.

The differentially expressed genes (DEGs) for each cell type between the control and heat-treated conditions were detected when there was a |log2FC| ≥ 0.36 difference in the average expression level and when *P* < 0.05 (Additional file 10: Table S9). We found that the DEGs varied in different cell types: mesophyll cells (5924 up, 2143 down) > epidermal cells (4030 up, 1212 down) > vascular cells (2368 up, 934 down) > proliferating cells (999 up, 426 down) > guard cells (770 up, 246 down) (Fig. 3c). Of them, the expression of only 150 upregulated DEGs and 92 downregulated DEGs was altered in all five cell types (Fig. 3d). In contrast, more DEGs were specifically active in a single cell type, including 3202 in mesophyll cells (2261 up, 941 down), 906 in epidermal cells (610 up, 296 down), 457 in vascular cells (297 up, 160 down), 235 in proliferating cells (120 up, 115 down), and 108 in guard cells (73 up, 35 down). These cell type-specific DEGs were enriched in various GO terms and were also different from those in the overall leaf tissues (Additional file 1: Figure S5). Cell type-specific DEGs in the epidermis, the outermost cell layer surrounding the leaves, were mostly enriched in the categories “ribosome,” “channel activity,” and “stress regulation” in epidermal cells and “sucrose” in guard cells. In mesophyll cells and proliferating cells, DEGs showed signatures for “organophosphate,” “histone,” “amino acids catabolic,” “kinase,” and “cellular and intercellular.” In vascular cells, the DEGs were predicted to be significantly enriched in “transport” processes.

### Cell type-specific heat stress response marker genes

Expression analysis of the cell type-specific DEGs whose expression changed after heat treatment only in one cell type could be considered potential heat stress response marker genes for different cell populations (Additional file 11: Table S10; Additional file 1: Figure S5). For example, *BAA03g00780*, which is syntenic to *Arabidopsis AT5G02380* (*MT2B*) and encodes a small, cysteine-rich metal-binding protein active in the response to lead exposure, NaCl stress, and heavy metal tolerance, was upregulated in mesophyll cells after heat treatment; *BAA03g43520*, which is syntenic to the *Arabidopsis* salt stress response gene *AT3G52590* (*UBQ1*), was upregulated in epidermal cells after heat treatment; and *BAA09g53920*, which is syntenic to *Arabidopsis AT3G62550*, which encodes a protein with an ATP-binding motif, was downregulated in mesophyll cells. Moreover, many *Brassica* genes with unknown functions, such as *BAA09g04920* in epidermal cells, *BAA07g19420* in vascular cells, *BAA03g14340* in guard cells, and *BAA10g23280* in proliferating cells, were specifically down- or upregulated in one cell type after heat treatment (Fig. 3e). These cell-specific corresponding genes with unknown functions need to be studied to explore the cell-specific thermal response mechanism.

### Expression patterns of HSPs in different cell types

There were more upregulated genes than downregulated genes in all five cell types after heat stress. By investigating heat-responsive genes at the cellular level, we found differences at the tissue level. The expression of HSP genes, which are well-known target genes of heat stress responsive TFs, was more often downregulated than upregulated under heat stress in this study. We found that high temperature downregulated 38 *HSP* genes and upregulated eight *HSPs* of various families. The expression of these HSPs was different in the different cell populations, but not all of them showed significant changes in a particular cell type. For downregulated *HSPs*, the expression of 23 *HSPs* was altered in all five cell types, while 15 HSPs showed a cell-type-specific response. For example, *HSP23.6* and *HSP90-7* were downregulated only in vascular cells; *HSP90-6* was downregulated only in epidermal cells; *HSP22* was downregulated in epidermal cells and vascular cells; and *HSP15.7* was downregulated in mesophyll cells and vascular cells. The eight upregulated *HSPs* all showed cell-type-specific expression changes. HSP90-5 and HSP70-16 were upregulated in only mesophyll cells, and HSP70-1 was upregulated in mesophyll cells and vascular cells.

There are multiple copies of HSPs in Chinese cabbage, and we found that duplicated pairs of genes exhibit differences in expression profiles in response to heat stress in the same cell type. Three orthologous genes of HSP18.2 were identified between Chinese cabbage (LF-*BAA10g17490*, MF1-*BAA03g11920*, MF2-*BAA02g11630*) and *Arabidopsis* (*AT5G59720*). The expression of HSP18.2 was not affected by heat stress in the mesophyll cells, epidermal cells, guard cells, or proliferating cells. In the vascular cells, MF2-*BAA02g11630* expression increased, whereas LF-*BAA10g17490* and MF1-*BAA03g11920* expression decreased. For the genes present in multiple copies in the Chinese cabbage LF, MF1, and MF2 subgenomes, differential expression patterns were also detected. Collectively, the genes whose expression was affected by heat stress were more highly expressed in LF than in MF1 and MF2 (Fig. 3f).

### Heat stress resulted in opposite gene expression patterns in different cell types

The heat stress response is essentially a single-cell response. The pattern of gene expression in different cell types under heat stress conditions is different. In particular, some genes showed opposite expression patterns in different cell types in response to heat stress (Additional file 12: Table S11). We found 43 genes in the mesophyll cells, 140 genes in the epidermal cells, 97 genes in the guard cells, 65 genes in the vascular cells, and 54 genes in the proliferating cells that showed opposite expression patterns in the other cell types (Additional file 1: Figure S6). For example, the calcium-binding EF-hand family protein-encoding gene MSS3 was upregulated in mesophyll cells but downregulated in the epidermal cells after heat treatment; the gene encoding a WRKY DNA-binding protein, WRKY8, was upregulated in the vascular cells but downregulated in the mesophyll cells; and many ribosomal protein family genes, including S8e, L2, L22e, L24e, and L36e family members, were upregulated in the epidermal cells but downregulated in the other cell types (Fig. 3g). These observations reveal the divergence of the expression of duplicated genes and highlight a new perspective for studying gene functional diversification.

## Discussion

This study exploited the Drop-Seq method to profile transcriptomes in diverse types of cells originated from Chinese cabbage shoots and young leaves. We used two biological replicates for each kind of samples, collected more than 30,000 individual cells, included the 6 major cell types, and obtained single-cell transcriptomic data sets on these cells. Three layers of information were revealed in this study: cell type-specific differential gene expression, subgenome dominance in different cell types, and cell type-specific heat response activity in leaves.

First, cell type-specific differential gene expression was revealed to help understand heterogeneity among leaf cells. Chinese cabbage has a complex morphology with variable leaf shape, and optimization of leaf morphology is important for Chinese cabbage yield and marketability. Our scRNA-seq data provide insights into Chinese cabbage leaf development. The mesophyll cell, epidermal cell, guard cell, vascular cell, and proliferating cell marker genes we characterized could be used in studies leaf development involving *Brassica* species.

Second, we discovered the link between expression-level dominance and cell types with respect to the mesotriplication of the A genome in *B. rapa*. Genome polyploidization is frequent and widespread in plants; polyploidization results in an abundance of gene family expansion and produces genetic or phenotypic variations to support plants for better adaptation to different environments [32, 33]. The differentiation among subgenomes can be observed from both the gene density and gene expression levels [34]. In *B. rapa*, the LF subgenome retained more genes and contributed more highly expressed genes in different tissues; thus, the LF subgenome has been the dominant subgenome since WGT [35]. In two *B. rapa* accessions (Chiifu subspecies *pekinensis* and L58 subspecies *parachinensis*), the genes in the LF subgenome were dominantly expressed over the genes in the MF subgenomes (MF1 and MF2), while the genes in MF1 were slightly dominantly expressed over the genes in MF2 in different organs, including leaf, stem, and root [36]. Consistent with this, Chinese cabbage gene expression is biased in favor of the LF subgenome, which contributed a high number of expressed genes. Along with drastic genome changes, many Chinese cabbage genes had no syntenic orthologs in *Arabidopsis*, which we grouped into UG. The number of genes in this group was the largest, but the number of genes expressed in this group was very low. In addition, the relative proportions of the mRNA transcripts are balanced in different cell types. The influence of dose balance with respect to gene expression is expected to be important for successful function [37]. We found that since the WGT, the genes in Chinese cabbage have been maintained in a certain balance not only at the tissue level but also at the cellular level in different cell types. Moreover, genes present in multiple copies may also be functionally different. Novel functions (neofunctionalization) or split functions (subfunctionalization) result from changes in the gene expression of one or both of the genes present in multiple copies, resulting in differences in gene function [38]. Few data are available on this topic, especially in specific cell types at the whole-genome level. We observed variations in the expression patterns of multicopy genes among cell types and entry points to study functional differentiation during the formation of different cell types.

Third, by comparing cells from leaves grown under heat stress, we discovered that heat stress does not substantially alter cell type identity but does lead to changes in the relative proportions of cell-type-specific gene expression. Heterogeneity in the gene expression response during heat stress was observed among cell types; although some genes had similar gene expression patterns, the majority of genes responded to stress in a cell-type-specific manner. More DEGs were identified in mesophyll cells, vascular cells, epidermal cells, and guard cells than in the other cell types, while fewer were identified in proliferating cells. This result indicates that cell-type-specific expression changes often took place in differentiated cells, consistent with previous observations reported in many abiotic stress experiments, such as those involving heat-shock-treated *Arabidopsis* roots and low-nitrogen- and high-salinity-treated rice seedlings [36, 39]. In addition to the differences in the numbers of DEGs and gene enrichment functions of the heat-responsive genes among cell types, there were also cell type differences in the relationships between multiple-copy genes in Chinese cabbage. We found the same functional enrichment of differentially expressed genes in Chinese cabbage leaf cells and *Arabidopsis* root cells upon heat shock. Heat stress not only affects gene expression in different tissues but also has an impact in a cell-type-specific manner, as shown by the different expression patterns of the HSP family members among leaf cell types during heat stress. Genes that were strongly induced by heat stress showed a difference in their expression at the whole plant and tissue levels, and more genes were differentially expressed in response to heat stress across the different cell types. Given that research on abiotic resistance has now advanced to the cellular level, we have discovered some cell-specific heat-corresponding marker genes that could be used to identify plant heat responses in different cells to stimulate future heat stress studies. All these DEGs in the three subgenomes were screened in the KEGG database for pathway annotation in different cell types (Additional file 1: Figure S6). Within the same cell type, the enrichment function of DEGs differed among different subgenomes. For example, DEGs in LF were enriched in the fatty acid metabolism pathway, which is linked to plant temperature stress responses, but this pathway was not enriched in MF1 and MF2. This result indicates that functional differentiation of multicopy genes in the three subgenomes exists in the heat stress response regulatory network.

WGT contributes to evolutionary innovation and increases the appearance of novel traits as well as potential genetic redundancy. Following WGT events, *Brassica* regulatory networks might have diverged and rewired, depending on the mode of triplication and functional category. To increase our knowledge of how the network evolves subsequent to such events, the expression data of regulatory genes involved in the heat stress response pathway were investigated (Additional file 1: Figure S7). We found that duplicate genes display greater levels of diversity in their expression in response to heat processes. This diversity was found in different cell types and between subgenomes. *GROWTH REGULATING FACTOR 7* (*GRF7*) duplicates in LF were upregulated in epidermal cells, mesophyll cells, vascular cells, and proliferating cells, while in MF2, they were upregulated only in mesophyll cells. Many genes in MF1 and MF2 were induced/decreased, but the genes in LF, such as *CNGSs*, *DREB2A*, and *HsfA2*, did not show differential expression under heat stress. Although some examples show that expression of duplicate genes in two subgenomes is affected by heat stress, such as in MF1 and MF2, there are more genes that respond to heat in the major subgenome LF. The redundancy of essential genes not only increases organism robustness and selective advantages but also constrains rewiring of plant regulatory networks. The heat stress response is fully integrated with the physiological stress response and should be considered a component of a system-wide gene network coordinated across a variety of cells and tissues. Because environmental factors tend to change faster than internal factors, the expression of genes that respond to biotic/abiotic stress (external) diverges faster than that of homeologs involved in developmental programs (internal) [40]. Many plants respond to environmental stresses by inducing expression of stress-related genes. In addition, the genes involved in developmental processes tend to be coregulated. For example, the expression patterns of the functionally redundant MADS-box genes *SEP1/2/3*, which are involved in flower development, are correlated [41]. Single knockout of one gene showed no developmental defect [42]. In contrast, genes involved in biotic/abiotic stresses tend to show divergent expression patterns. For example, the expression levels of cyclophilin genes, or *CYPs*, in which some duplicates are induced by biotic/abiotic stress, differed greatly in Fava Bean [43]. *bZIP28*, encoding a putative membrane-tethered transcription factor, was upregulated in response to heat. *bZIP28*-LF and *bZIP28*-MF1 are duplicated genes expressed in proliferating cell and mesophyll cell. However, *bZIP28*-LF was upregulated, and *bZIP28*-MF1 was downregulated under heat stress in this study, suggesting that the duplicated genes may be involved in different regulatory networks. Our study focuses on divergence in gene expression and takes into account regulatory and functional divergence at the cell type level.

## Conclusions

Overall, our scRNA-seq analyses of Chinese cabbage highlights the existence of diverse transcriptional networks in different leaf cell types, and the correlations between the expression-level dominance of subgenomes and dynamic cell type-specific transcriptional landscapes with leaf responses to heat stress. These findings help to illustrate the transcriptomic atlas during leaf development, promote the understanding of leaf physiology at single-cell resolution, and investigate the maintenance of multiple-copy genes in other species. These findings can also facilitate to characterize the effects of heat stress at the single-cell level, breed better crop plants with improved high-temperature tolerance, and markedly improve cropping systems.

## Methods

### Plant materials and growth conditions

Inbreeding line *B. rapa* ssp. *pekinensis* (Chinese cabbage A03) seedlings were grown in soil under 25 °C day/18 °C night and 16 h light/8 h dark conditions in a climate-controlled growth chamber. For the heat treatment group, the plants were transferred to 40 °C for 12 h, and protoplasts were harvested immediately afterward together with those of control plants, which were allowed to keep growing at 21/16 °C.

### Protoplast isolation

Shoot apices of 1-week-old plants and second true leaves of 4-week-old plants from both the control and heat-stressed groups were used for protoplast harvesting. Thirty-five shoot apices were digested in RNase-free enzyme solution #1 (1.5% cellulase R10, 1.5% macerozyme R10, 0.4 M mannitol, 0.1 M 4-morpholineethanesulfonic acid, 10 mM KCl, 10 mM CaCl_2_, and 0.1% bovine serum albumin (BSA); pH 5.7) for 2 h at 30 °C on a shaker at 50 revolutions/minute. Similarly, twelve young leaves were digested in RNase-free enzyme solution #2 (1.5% cellulase R10, 0.75% macerozyme R10, 0.6 M mannitol, 10 mM CaCl_2_, 20 mM KCl, 20 mM MgCl_2_ and 0.1% BSA; pH 5.7) for 2 h at 27 °C on a shaker at 45 revolutions/minute. The protoplasts were filtered 1 time through a 100 mm cell strainer and 2 times through a 40-mm cell strainer and then centrifuged at 100×*g* for 5 min at 4 °C. The supernatant was gently removed and washed 2 times with wash solution (0.5 M mannitol and 0.04% BSA). After filtering the supernatant through a 30 mm cell strainer, protoplast viability was determined by 0.4% trypan blue staining, and the concentration was determined by a hemocytometer. The ratio of viable cells to total cells of each sample was higher than 95%. The concentration of protoplasts was ~700–1000 cells/μL and adjusted to 60 cells/μL for further processing.

### Microscopy

One-week-old shoots and 4-week-old leaves were harvested and fixed with 5% glutaraldehyde in 0.1 M buffer for 24 h at 4 °C. The tissues were dehydrated in 10%, 30%, 50%, 70%, 95% and 100% ethanol treatments; cleared with an ethanol:xylene (1:1) solution and xylene for 1 h; and then embedded with Paraplast. Waxed sections were cut to 7 μm, stained with 5% toluidine blue for 30 s, washed three times with water, and deparaffinized with xylene. The sections were then observed by microscopy.

In addition, the 2-week-old shoot apices were also scanned by electron microscopy. The tissues were fixed with 4% glutaraldehyde overnight at room temperature; dehydrated in 30%, 70%, 90%, and 100% ethanol treatments; and then dried for 6 h. The samples were coated with gold powder and observed by microscopy.

### scRNA-seq library construction and sequencing

We prepared an scRNA-seq library from two biological replicates of shoots and from second true leaves of plants in the control and heat treatment groups. For each replicate, the protoplast suspensions were loaded into a 10x Genomics GemCode single-cell instrument to generate single-cell gel beads in emulsion using a Chromium Single Cell 3’ Reagent Kit v3. Barcoded cDNA amplification was performed with temperature cycles of 45 min at 53 °C and 5 min at 85 °C. Single-cell RNA-seq libraries were prepared using a Chromium Single Cell 30 Gel Bead and Library Kit. The library qualities were checked by an Agilent 2100 Bioanalyzer, and the concentration of the libraries was measured by Qubit (Invitrogen). The scRNA-seq libraries were ultimately sequenced with an Illumina HiSeq 4000 instrument.

### Data analysis

#### Preprocessing

Sequencing reads of the six scRNA-seq samples (S-C1, S-C2, L-C1, L-C2, L-H1 and L-H2) were aligned to the Chinese cabbage A03 v1 reference genome (www.bioinformaticslab.cn/EMSmutation/home) by the 10X Genomics Cell Ranger Pipeline (v5.0.0) with the default parameters (http://support.10xgenomics.com/single-cell/software/overview/welcome). Low-quality reads were removed, and the remaining reads were uniquely mapped to the transcriptome. Reads covering at least 50% of an exon were considered for UMI counting by Star (https://github.com/alexdobin/STAR). UMI counting and cell barcode calling produced the cell-by-gene matrices, and the cell-by-gene matrices for each sample were individually imported into the Seurat v3.1.1 package for downstream analysis [44].

#### Cell clustering, annotation, and marker gene selection

Before downstream analysis, we removed the cells with UMIs greater than 50,000 and lower than 500 using EmptyDrops, removed probable doublets using DoubletFinder (v.2.0.2), and removed cells in which the numbers of expressed genes were less than 200 and greater than 10,000 [45, 46]. Gene expression for each cell was normalized by the “LogNormalize” method. The effects of batch effects and behavioral conditions on clustering were minimized by Harmony [47].

For cell clustering, the Louvain method was used [48]. For cell cluster visualization, t-SNE and UMAP were performed using the same proliferating cells [49]. During these analyses, 0.7 resolution was used for two groups of sample combinations. The number of cells from shoot and leaf samples was counted in each cluster, and the cell enrichment between shoot and leaf samples was statistically analyzed by Fisher’s test. In addition, the total number of cells in each sample was normalized to 10,000 cells and then used to draw the bar charts for each cell cluster.

The cell type of each cluster was defined by the following methods: (1) defined by known marker genes in Additional file 6: Table S5; (2) correlated between individual clustered cells; and (3) defined by the top, well-characterized cluster-enriched genes. Genes were considered enriched in clusters when their expression value in the target cluster was 1.28-fold (log_2_FC > 0.36) greater than that in the other clusters and when their minimum fraction was at least 0.25. After cell type annotations, the novel marker genes for each cell type were identified according to a log_2_FC > 0.5 and *P* ≤0.01, and the genes needed to be expressed in 25% of cells of the target type and in < 25% of cells of all the other types.

#### Comparison of predominantly expressed genes across the three subgenomes

Syntenic *B. rapa* genes in *Arabidopsis* were determined by both sequence similarity and collinearity of flanking genes using the method reported by Cheng [3]. Syntenic paralogous pairs (one-to-one, one-to-two, and one-to-three copies) were extracted. Genes expressed in at least 5% of cells of the target type were counted. One to three copies of genes were used to analyze the predominant gene expression. The number of predominantly expressed genes had a |log2FC|≥0.36 and a *p* value of ≤0.05 between the LF and MF subgenomes and between the MF1 and MF2 subgenomes in each cell type.

#### Comparison of DEGs after heat stress and cell-type-specific heat response marker gene identification

The expression value of each gene in different cell types was compared against the rest of the cells using the MAST approach [50]. To carry out accurate gene expression profiling, the expression of reference genes in different cell types between the control and treatment groups was analyzed in different cell types (Additional file 9: Table S8). Genes whose log2FC in expression was ≥0.36 in the target cell type, genes that were expressed in more than 25% of the cells belonging to the target cell type and genes whose *p* value was less than 0.05 were considered differentially expressed between the control and heat stress cells. Genes that were differentially expressed (upregulated or downregulated) in only one cell type were identified as heat stress response marker genes. The marker genes among cell types were visualized by UMAP plots using the function “upset” in the R package UpSetR (v1.4.0) [51].

#### Gene functional enrichment and pathway analysis

DEGs identified in this study were subjected to GO and KEGG enrichment analyses [52, 53]. The top 50 enrichment pathways with high significance were annotated and used to construct an enrichment heatmap for the different cell types.

## Supplementary Information

Additional file 1: Figure S1-S7.

Additional file 2: Table S1. Summary of the cell data in Chinese cabbage vegetative shoot apex and young leaf samples before and after filtering.

Additional file 3: Table S2. Cluster information for 19 cell clusters of Chinese cabbage vegetative shoot apex and young leaf cells.

Additional file 4: Table S3. Cluster-enriched gene list and gene descriptions for 19 cell clusters.

Additional file 5: Table S4. Gene Ontology and Kyoto Encyclopedia of Genes and Genomes analyses for cluster-enriched genes in 19 cell clusters.

Additional file 6: Table S5. Marker genes involved in the identification of cell types.

Additional file 7: Table S6. Candidate cell type marker genes identified in Chinese cabbage.

Additional file 8: Table S7. Predominantly expressed genes in subgenomes in different cell types.

Additional file 9: Table S8. The expression of reference genes in different cell types between the control and treatment groups.

Additional file 10: Table S9. Differentially expressed genes (DEGs) for each cell type between the control and heat conditions.

Additional file 11: Table S10. Gene expression changed after heat treatment in only one cell type.

Additional file 12: Table S11. Different genes showed opposite expression patterns in different cell types under heat stress conditions.

Additional file 13. Review history.

## Data Availability

The raw sequence data reported in this paper have been deposited in the Genome Sequence Archive in National Genomics Data Center, China National Center for Bioinformation/Beijing Institute of Genomics, Chinese Academy of Sciences (GSA: CRA008834) that are publicly accessible at https://ngdc.cncb.ac.cn/gsa [54].

## References

[1] Du F, Guan C, Jiao Y (2018). Molecular mechanisms of leaf morphogenesis. Mol Plant.

[2] Gonzalez N, Vanhaeren H, Inzé D (2012). Leaf size control: complex coordination of cell division and expansion. Trends Plant Sci.

[3] Cheng F, Wu J, Fang L, Wang X (2012). Syntenic gene analysis between *Brassica rapa* and other *Brassicaceae* species. Front Plant Sci.

[4] Kalve S, De Vos D, Beemster GT (2014). Leaf development: a cellular perspective. Front Plant Sci.

[5] Tang F, Barbacioru C, Wang Y, Nordman E, Lee C, Xu N (2009). mRNA-Seq whole-transcriptome analysis of a single cell. Nat Methods.

[6] Zhang TQ, Chen Y, Wang JW (2021). A single-cell analysis of the *Arabidopsis* vegetative shoot apex. Dev Cell.

[7] Liu H, Hu D, Du P, Wang L, Liang X, Li H (2021). Single-cell RNA-seq describes the transcriptome landscape and identifies critical transcription factors in the leaf blade of the allotetraploid peanut (*Arachis hypogaea* L.). Plant Biotechnol J.

[8] Allakhverdiev SI, Kreslavski VD, Klimov VV, Los DA, Carpentier R, Mohanty P (2008). Heat stress: an overview of molecular responses in photosynthesis. Photosynth Res.

[9] Yang D, Peng S, Wang F (2020). Response of photosynthesis to high growth temperature was not related to leaf anatomy plasticity in rice (*Oryza sativa* L.). Front Plant Sci.

[10] Oh S, Moon KH, Son IC, Song EY, Moon YE, Koh SC (2014). Growth, photosynthesis and chlorophyll fluorescence of Chinese cabbage in response to high temperature. Horticultural Sci Technol.

[11] Ohama N, Sato H, Shinozaki K, Yamaguchi-Shinozaki K (2017). Transcriptional regulatory network of plant heat stress response. Trends Plant Sci.

[12] Jean-Baptiste K, McFaline-Figueroa JL, Alexandre CM, Dorrity MW, Saunders L, Bubb KL (2019). Dynamics of gene expression in single root cells of Arabidopsis thaliana. Plant Cell.

[13] Zondlo SC, Irish VF (1999). CYP78A5 encodes a cytochrome P450 that marks the shoot apical meristem boundary in *Arabidopsis*. Plant J.

[14] Cho E, Zambryski PC (2011). ORGAN BOUNDARY1 defines a gene expressed at the junction between the shoot apical meristem and lateral organs. Proc Natl Acad Sci.

[15] Tian C, Wang Y, Yu H, He J, Wang J, Shi B (2019). A gene expression map of shoot domains reveals regulatory mechanisms. Nat Commun.

[16] Pedersen DS, Coppens F, Ma L, Antosch M, Marktl B, Merkle T (2011). The plant-specific family of DNA-binding proteins containing three HMG-box domains interacts with mitotic and meiotic chromosomes. New Phytol.

[17] Park M, Krause C, Karnahl M, Reichardt I, El Kasmi F, Mayer U (2018). Concerted action of evolutionarily ancient and novel SNARE complexes in flowering-plant cytokinesis. Dev Cell.

[18] Guo Y, Qin G, Gu H, Qu LJ (2009). *Dof5. 6/HCA2*, a Dof transcription factor gene, regulates interfascicular cambium formation and vascular tissue development in Arabidopsis. Plant Cell.

[19] Cayla T, Batailler B, Le Hir R, Revers F, Anstead JA, Thompson GA (2015). Live imaging of companion cells and sieve elements in *Arabidopsis* leaves. PLoS One.

[20] Wang F, Muto A, Van de Velde J, Neyt P, Himanen K, Vandepoele K, Van Lijsebettens M (2015). Functional analysis of the *Arabidopsis TETRASPANIN* gene family in plant growth and development. Plant Physiol.

[21] Zhu Y, Liu L, Shen L, Yu H (2016). NaKR1 regulates long-distance movement of FLOWERING LOCUS T in *Arabidopsis*. Nat Plants.

[22] Abe M, Takahashi T, Komeda Y (2001). Identification of a cis-regulatory element for L1 layer-specific gene expression, which is targeted by an L1-specific homeodomain protein. Plant J.

[23] Sasaki T, Mori IC, Furuichi T, Munemasa S, Toyooka K, Matsuoka K (2010). Closing plant stomata requires a homolog of an aluminum-activated malate transporter. Plant Cell Physiol.

[24] Negi J, Moriwaki K, Konishi M, Yokoyama R, Nakano T, Kusumi K (2013). A Dof transcription factor, SCAP1, is essential for the development of functional stomata in *Arabidopsis*. Curr Biol.

[25] Takada S, Takada N, Yoshida A (2013). ATML1 promotes epidermal cell differentiation in *Arabidopsis* shoots. Development.

[26] Matos JL, Lau OS, Hachez C, Cruz-Ramírez A, Scheres B, Bergmann DC (2014). Irreversible fate commitment in the Arabidopsis stomatal lineage requires a FAMA and RETINOBLASTOMA-RELATED module. Elife.

[27] Liu Z, Zhou Y, Guo J, Li J, Tian Z, Zhu Z (2020). Global dynamic molecular profiling of stomatal lineage cell development by single-cell RNA sequencing. Mol Plant.

[28] Salesse C, Sharwood R, Sakamoto W, Stern D (2017). The rubisco chaperone BSD2 may regulate chloroplast coverage in maize bundle sheath cells. Plant Physiol.

[29] Sun X, Li X, Lu Y, Wang S, Zhang X, Zhang K (2022). Construction of a high-density mutant population of Chinese cabbage facilitates the genetic dissection of agronomic traits. Mol Plant.

[30] Chandna R, Augustine R, Bisht NC (2012). Evaluation of candidate reference genes for gene expression normalization in *Brassica juncea* using real time quantitative RT-PCR. PLoS One.

[31] Ma L, Wu J, Qi W, Coulter JA, Fang Y, Li X (2020). Screening and verification of reference genes for analysis of gene expression in winter rapeseed (*Brassica rapa* L.) under abiotic stress. PLoS One.

[32] Bowers JE, Chapman BA, Rong J, Paterson AH (2003). Unravelling angiosperm genome evolution by phylogenetic analysis of chromosomal duplication events. Nature.

[33] Hittinger CT, Carroll SB (2007). Gene duplication and the adaptive evolution of a classic genetic switch. Nature.

[34] Schnable JC, Springer NM, Freeling M (2011). Differentiation of the maize subgenomes by genome dominance and both ancient and ongoing gene loss. Proc Natl Acad Sci.

[35] Cheng F, Sun C, Wu J, Schnable J, Woodhouse MR, Liang J (2016). Epigenetic regulation of subgenome dominance following whole genome triplication in *Brassica rapa*. New Phytol.

[36] Cheng F, Wu J, Fang L, Sun S, Liu B, Lin K (2012). Biased gene fractionation and dominant gene expression among the subgenomes of *Brassica rapa*. PLoS One.

[37] Birchler JA, Veitia RA (2012). Gene balance hypothesis: connecting issues of dosage sensitivity across biological disciplines. Proc Natl Acad Sci.

[38] Panchy N, Lehti-Shiu M, Shiu SH (2016). Evolution of gene duplication in plants. Plant Physiol.

[39] Wang Y, Huan Q, Li K, Qian W. Single-cell transcriptome atlas of the leaf and root of rice seedlings. J Genet Genomics. 2021;48(10):881–98. 10.1016/j.jgg.2021.06.001 34340913

[40] Ha M, Li WH, Chen ZJ (2007). External factors accelerate expression divergence between duplicate genes. Trends Genet.

[41] Jack T (2001). Relearning our ABCs: new twists on an old model. Trends Plant Sci.

[42] Pelaz S, Ditta GS, Baumann E, Wisman E, Yanofsky MF (2000). B and C floral organ identity functions require *SEPALLATA MADS-box* genes. Nature.

[43] Luan S, Lane WS, Schreiber SL (1994). pCyP B: a chloroplast-localized, heat shock-responsive cyclophilin from fava bean. Plant Cell.

[44] Butler A, Hoffman P, Smibert P, Papalexi E, Satija R (2018). Integrating single-cell transcriptomic data across different conditions, technologies, and species. Nat Biotechnol.

[45] Lun AT, Riesenfeld S, Andrews T, Gomes T, Marioni JC (2019). EmptyDrops: distinguishing cells from empty droplets in droplet-based single-cell RNA sequencing data. Genome Biol.

[46] McGinnis CS, Murrow LM, Gartner ZJ (2019). DoubletFinder: doublet detection in single-cell RNA sequencing data using artificial nearest neighbors. Cell Syst.

[47] Korsunsky I, Millard N, Fan J, Slowikowski K, Zhang F, Wei K (2019). Fast, sensitive and accurate integration of single-cell data with Harmony. Nat Methods.

[48] Rotta R, Noack A (2011). Multilevel local search algorithms for modularity clustering. J Exp Algorithmics.

[49] Van der Maaten L, Hinton G (2012). Visualizing non-metric similarities in multiple maps. Mach Learn.

[50] Finak G, McDavid A, Yajima M, Deng J, Gersuk V, Shalek AK (2015). MAST: a flexible statistical framework for assessing transcriptional changes and characterizing heterogeneity in single-cell RNA sequencing data. Genome Biol.

[51] Lex A, Gehlenborg N, Strobelt H, Vuillemot R, Pfister H (2014). UpSet: visualization of intersecting sets. IEEE Trans Vis Comput Graph.

[52] Ashburner M, Ball CA, Blake JA, Botstein D, Butler H, Cherry JM (2000). Gene ontology: tool for the unification of biology. Nat Genet.

[53] Kanehisa M, Goto S (2000). KEGG: Kyoto Encyclopedia of Genes and Genomes. Nucleic Acids Res.

[54] Sun X., Feng D., Liu M., Qin R., Li Y., Lu Y., Zhang X., Wang Y., Shen S., Ma W., Zhao J. (2022). Single-cell transcriptome reveals dominant subgenome expression and transcriptional response to heat stress in Chinese cabbage. Genome Sequence Archive (GSA: CRA008834), https://ngdc.cncb.ac.cn/gsa. 10.1186/s13059-022-02834-4 PMC9762029 36536447

