# Supplementary material for: Single-cell transcriptome reveals dominant subgenome expression and transcriptional response to heat stress in Chinese cabbage
Source: Genome Biol. 2022 Dec 19;23:262. doi: 10.1186/s13059-022-02834-4 (PMC9762029; doi:10.1186/s13059-022-02834-4)
Supplement: Supplementary file 1 — Additional file 1: Figure S1-S7. [file 13059_2022_2834_MOESM1_ESM.pdf]

a

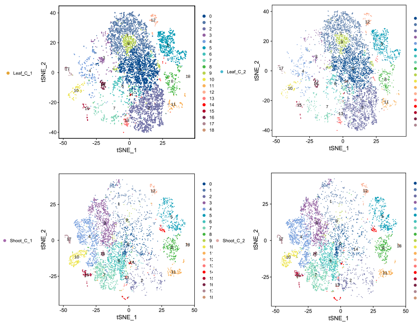

b

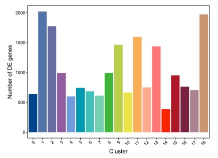

Cluster	Number of DE genes	Cluster	Number of DE genes
0	640	10	664
1	2021	11	1596
2	1775	12	749
3	991	13	1438
4	600	14	387
5	743	15	952
6	685	16	762
7	611	17	705
8	993	18	1973
9	1464		

c

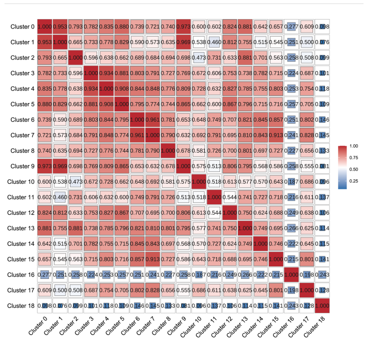

d

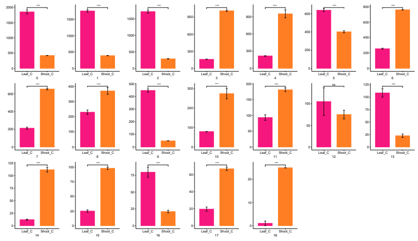

Cluster	Leaf_C_1	Leaf_C_2	Shoot_C_1	Shoot_C_2	Leaf_error	Shoot_error	p.value
0	1784	1919	421	417	67.5	2	1.39E-123
1	1722	1791	383	392	34.5	4.5	3.64E-121
2	1671	1765	280	294	47	7	2.01E-155
3	162	155	998	1024	3.5	13	4.41E-191
4	210	222	882	1022	6	70	1.66E-145
5	655	623	390	413	16	11.5	0.00206923
6	248	262	764	758	7	3	1.28E-85
7	200	224	663	645	12	9	4.79E-77
8	215	244	347	392	14.5	22.5	2.61E-18
9	434	460	48	49	13	0.5	1.17E-59
10	80	78	301	247	1	27	6.12E-38
11	84	102	187	175	9	6	1.14E-13
12	73	137	65	85	32	10	0.65030328
13	100	117	26	19	8.5	3.5	1.81E-10
14	11	13	107	116	1	4.5	1.22E-27
15	23	27	100	96	2	2	4.32E-16
16	87	72	19	23	7.5	2	2.46E-06
17	22	17	69	65	2.5	2	2.97E-10
18	2	0	25	25	1	0	1.43E-08

e

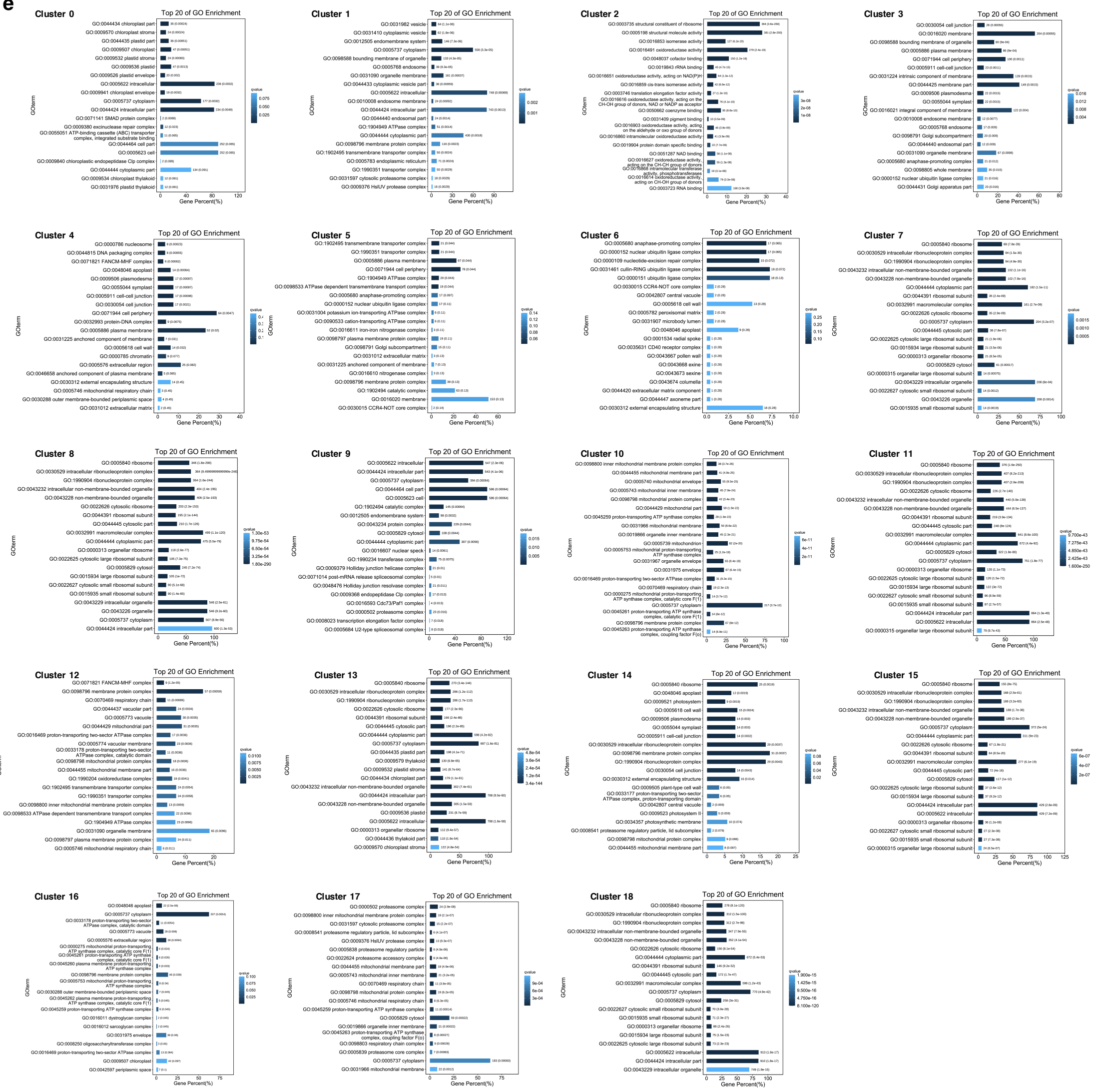

Figure S1. Information for Chinese cabbage shoot apices and developing leaf cell clusters. (a) t-SNE plots show the distribution of shoot and leaf cells for each replicate. (b)

Differentially expressed gene numbers in 19 cell clusters. (c) Correlations between 19 cell clusters. (d) Statistical analysis of cell enrichment between shoot and leaf samples in each cluster. Symbols indicate statistical significance from vehicle with p value thresholds of ≤0.05 (*), ≤0.01 (**) and ≤0.001 (***). (e) GO enrichment annotation of cellular components for genes in 19 cell clusters.

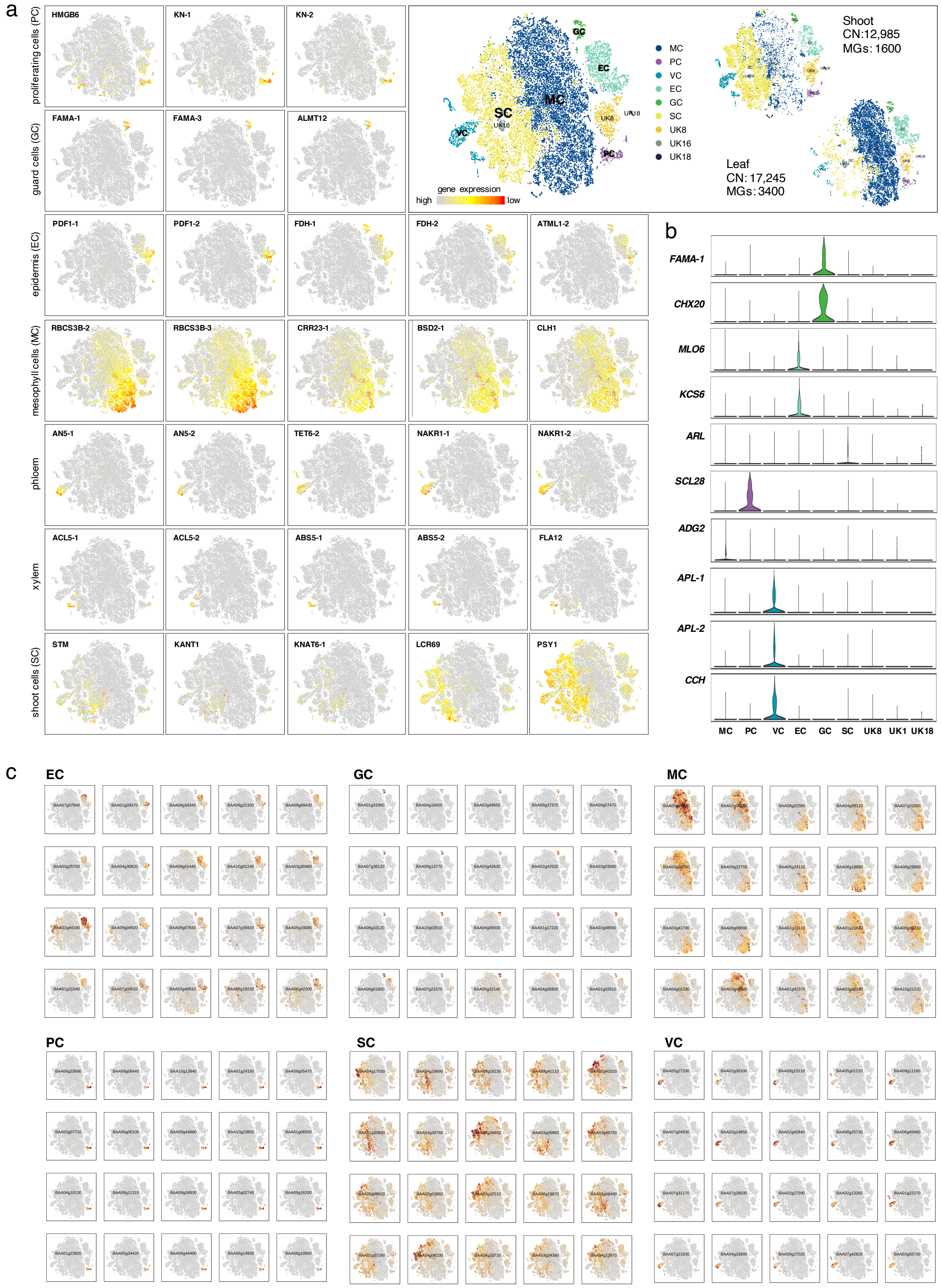

Figure S2. Marker genes for different cell types. (a) t-SNE plot showing the cell distribution of shoot and leaf samples and the expression distribution of selected marker genes. (b) Examples of novel cell-type marker genes for six major cell populations. (c) t-SNE plots

show the top 20 marker genes with the highest expression in each cell type in shoot and leaf samples.

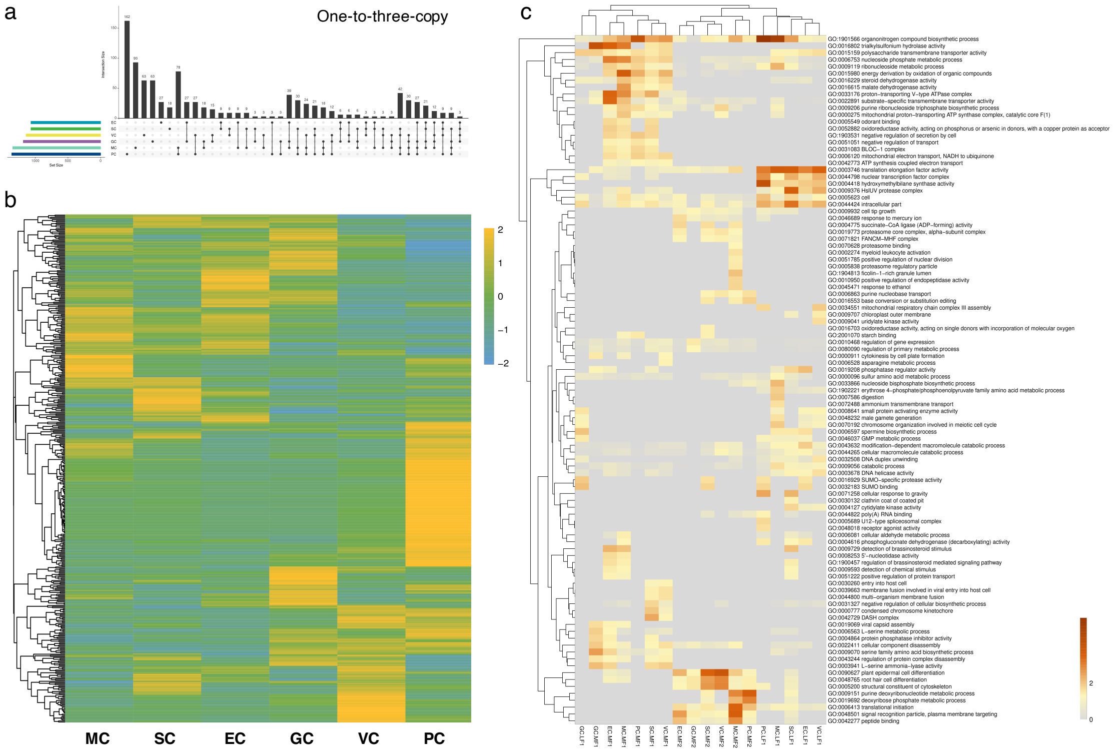

Figure S3. Predominantly expressed genes in three subgenomes. (a) Distribution of expressed one-to-three-copy genes in different cell types. (b) Heatmap for predominantly expressed genes in different cell types. (c) GO analysis of predominantly expressed genes in LF, MF1 and MF2 in different cell types.

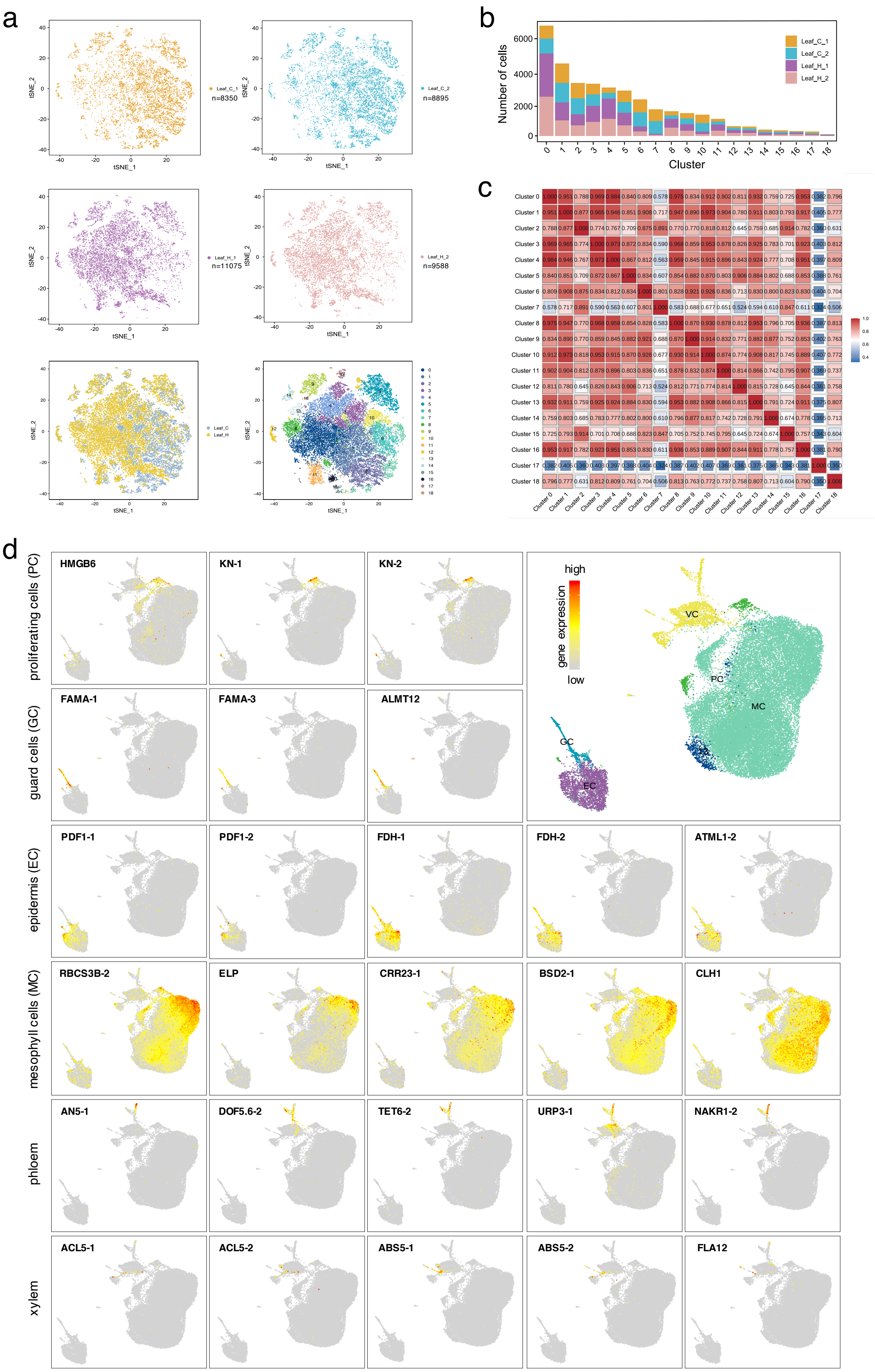
a
b
c
d
tSNE_1
tSNE_2
Leaf_C_1
n=8350
Leaf_C_2
n=8895
Leaf_H_1
n=11075
Leaf_H_2
n=9588
Leaf_C
Leaf_H
Number of cells
Cluster
Leaf_C_1
Leaf_C_2
Leaf_H_1
Leaf_H_2
proliferating cells (PC)
guard cells (GC)
epidermis (EC)
mesophyll cells (MC)
phloem
xylem
HMGB6
KN-1
KN-2
FAMA-1
FAMA-3
ALMT12
PDF1-1
PDF1-2
FDH-1
FDH-2
ATML1-2
RBCS3B-2
ELP
CRR23-1
BSD2-1
CLH1
AN5-1
DOF5.6-2
TET6-2
URP3-1
NAKR1-2
ACL5-1
ACL5-2
ABS5-1
ABS5-2
FLA12
high
gene expression
low
VC
PC
MC
GC
EC

Figure S4. Cell heterogeneity within Chinese cabbage leaves. (a) Distribution and numbers of cells for two biological replicates of leaf samples in the control and heat stress groups. (b) Cell numbers in 19 cell clusters. (c) Correlation between 19 leaf cell clusters. (d) t-SNE plot showing the cell distribution of leaf samples in the control and heat stress groups and the expression distribution of selected marker genes.

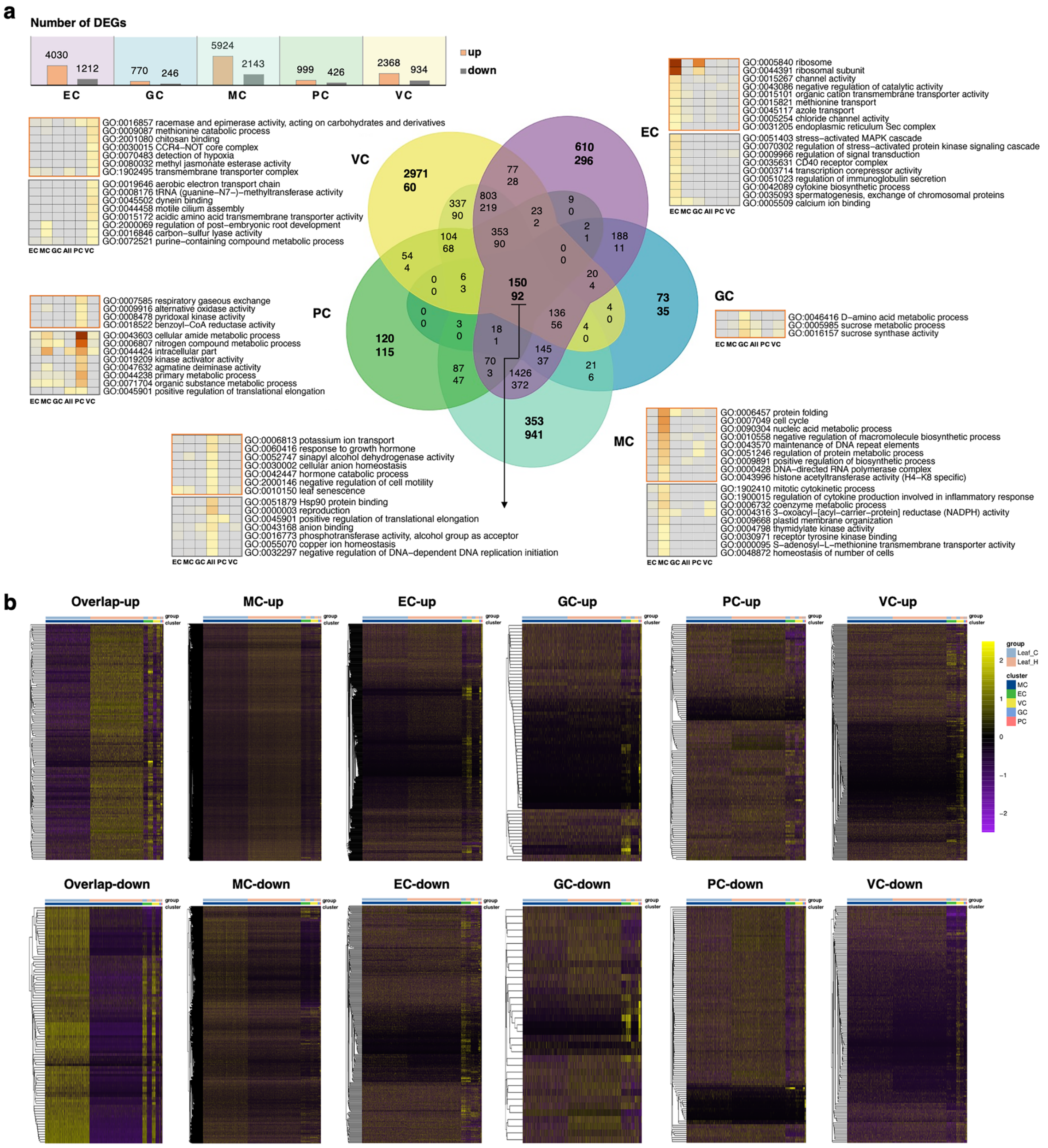

Figure S5. Differentially expressed genes (DEGs) for each cell type between the control and heat stress (HS) conditions. (a) Venn diagram for HS DEGs and GO enrichment analysis for cell-type-specific HS response genes. (b) Heatmaps for cell-type-specific HS

response marker genes showing changed expression patterns after heat treatment only in one cell type.

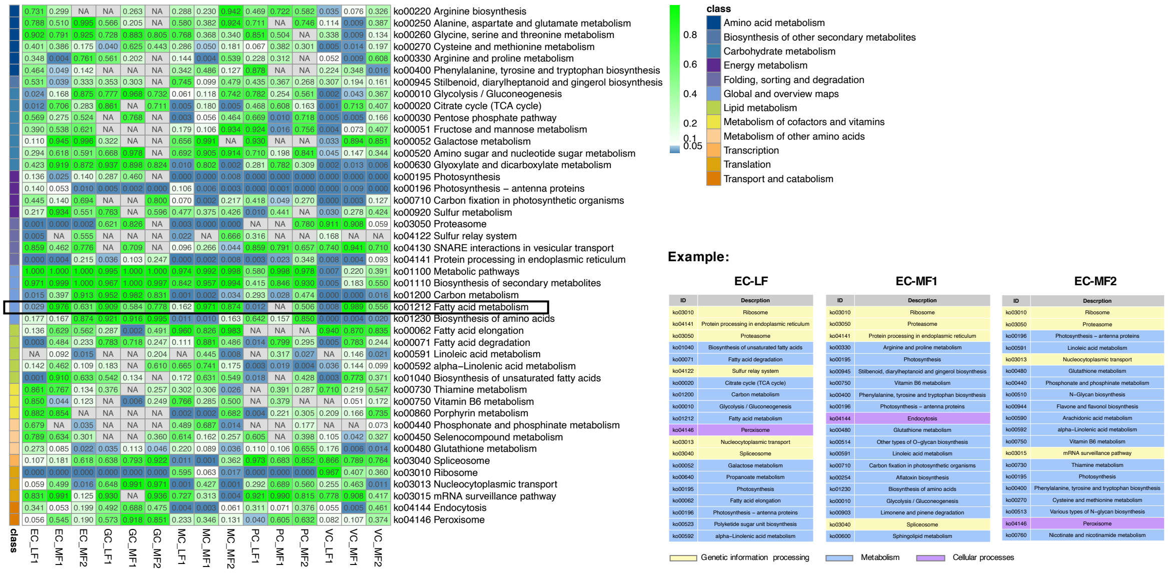

Figure S6. KEGG enrichment of differentially expressed genes across subgenomes in each cell type.

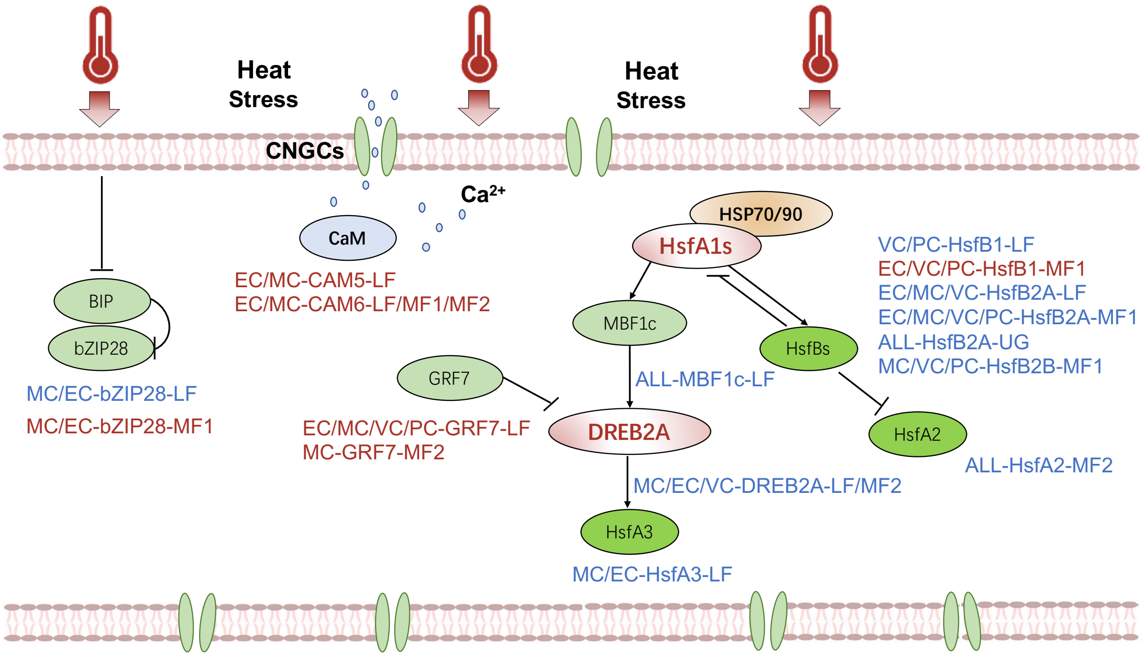

Figure S7. Expression overview of regulators involved in the heat stress response. Red indicates upregulation under HS, and blue indicates downregulation under HS.
