# Supplementary material for: Single-cell transcriptome reveals dominant subgenome expression and transcriptional response to heat stress in Chinese cabbage
Source: Genome Biol. 2022 Dec 19;23:262. doi: 10.1186/s13059-022-02834-4 (PMC9762029; doi:10.1186/s13059-022-02834-4)
Supplement: Supplementary file 13 — Additional file 13. Review history. [file 13059_2022_2834_MOESM13_ESM.docx]

Review History

**First round of review**

**Reviewer 1**

**Were you able to assess all statistics in the manuscript, including the appropriateness of statistical tests used?**

Yes, and I have assessed the statistics in my report.

**Comments to author:**

Review of the manuscript entitled "Single-cell transcriptome reveals dominant subgenome expression and the response to heat stress in Chinese cabbage" by Sun et al and submitted for publication to Genome Biology.

In this research manuscript, Sun et al. establish the transcriptome Chinese cabbage leaf and stem at the single-cell level by applying DROP-seq technology. Considering the Chinese cabbage as the product of a whole-genome triplication, the authors conducted a deeper analysis of each sub-genome to estimate their impact on the overall cellular transcriptome. In addition to developing this resource, the authors analyze the response of the leaf cells to high temperature, a major limitation to Chinese cabbage yields.

Overall, the authors generated quality data. However, additional analyses should be conducted to notably validate the impact of the heat stress on the plant, protoplast fitness, and transcriptome.

Major comments:

Line 107: The authors mentioned that they merged the shoot and leaf samples and analyzed 28,343 single-cell transcriptomes. However, the authors mentioned the analysis of 12,985 and 17,245 individual shoot and leaf cells, respectively. Could the authors explain this discrepancy?

Lines 112-114: Could the author conduct statistical analysis to support the significance of cell enrichment between shoot and leaf samples?

Lines 123-126: As written, this reviewer understands that all these marker genes are Chinese cabbage genes. However, later in the manuscript, the authors mostly cite references associated with Arabidopsis maize, peanut genes, etc. The use of orthologous (or homologous) genes should be explicitly mentioned. This reviewer was surprised that the use of this strategy between evolutionary distant plant species (e.g., chinese cabbage and maize) could support the functional annotation of the Chinese cabbage cells.

Lines 224-226: The authors identified syntenic orthologs according to sequence similarity. This is not a correct approach to defining syntenic orthologs. This should be done through the analysis of conservation of chromosomal segments based on their function, order, and direction on the chromosomes.

Lines 288-290: Could the authors provide control to support that the decrease in UMI number is a response to heat stress treatment and not to the fitness of the protoplast, an indirect consequence of the stress? For instance, could the author analyze the activity of reference genes that should not be differentially expressed between control and HS treatment? Such analysis is important to conduct to support the conclusion related to the DEG analysis in response to heat stress, and the identification of cell-type-specific heat stress marker genes. For instance, it was unexpected to find so many HSP genes down-regulated in response to heat stress (see lines 330-331). This reviewer also suggests the use of a specifically designed bioinformatics package to identify DEGs from single-cell RNA-seq datasets (e.g. DESingle; https://bioconductor.org/packages/release/bioc/html/DEsingle.html)

Figure 2 is another representation of Figure 1D. This reviewer suggests the deletion of Figure 2.

Overall, the resolution of the figures is very low (e.g., Figure 4 was almost impossible to read).

Minor comments:

Did the authors try to evaluate the induction of protoplastization-responsive genes to better estimate the quality of their clustering?

Line 121: Could the authors change this sentence as suggested: "…387 to 2,021 per cluster, and GO…"?

Line 200: Could the authors make the following change: "A03…" replaced by "The line Chinese cabbage A03 line…"?

Line 208: Please, remove "…in different cell types."

**Reviewer 2**

**Were you able to assess all statistics in the manuscript, including the appropriateness of statistical tests used?**

Yes, and I have assessed the statistics in my report.

**Comments to author:**

The authors reported a single-cell transcriptomic analysis to describe a cell atlas of Chinese cabbage leaves. The work identified 19 cell clusters, conducting a comprehensive annotation of six major cell types using known marker genes, and other novel marker genes for leaf development. This study provided new insight into studying the subgenomic evolution mechanism at the cellular level. In addition, this study underlies the mechanism on high temperature response in leaves by using the application of scRNA-seq technology. Followings are several minor comments.

Comments to author:

1. In material and method, the authors should describe more information on the analysis of gene expression differentiation among the subgenomes on single cell level. Particularly, it needs to know the criteria parameters used during the analysis.

2. Could the author compare the different expression patterns of multiple gene copies in different cell types with other studies? Maybe compared to other species or tissue-level transcriptome results in discussion.

3. In discussion (L407-411), the authors described the heat responses of different cell types in Arabidopsis roots. It is possible to find the same heat stress response patterns between roots and leaves on cellular level?

4. In Figure 4, genes names need to be italicized.

5. In L297, the number of down-regulated genes in VC is 1,077, but in Figure 4c is 934, please check it.

6. Please unify the writing format of subgenome names in the whole text and figures, "LF1" or "LF"?

7. The English writing of the article needs to be improved.

**Authors Response**

**Point-by-point responses to the reviewers’ comments:**

Reviewer #1:

Review of the manuscript entitled "Single-cell transcriptome reveals dominant subgenome expression and the response to heat stress in Chinese cabbage" by Sun et al and submitted for publication to Genome Biology. In this research manuscript, Sun et al. establish the transcriptome Chinese cabbage leaf and stem at the single-cell level by applying DROP-seq technology. Considering the Chinese cabbage as the product of a whole-genome triplication, the authors conducted a deeper analysis of each sub-genome to estimate their impact on the overall cellular transcriptome. In addition to developing this resource, the authors analyze the response of the leaf cells to high temperature, a major limitation to Chinese cabbage yields. Overall, the authors generated quality data. However, additional analyses should be conducted to notably validate the impact of the heat stress on the plant, protoplast fitness, and transcriptome.

*Response: Thank you for providing constructive and insightful comments. Please see detailed responses to your comments below.*

Major comments:

1. Line 107: The authors mentioned that they merged the shoot and leaf samples and analyzed 28,343 single-cell transcriptomes. However, the authors mentioned the analysis of 12,985 and 17,245 individual shoot and leaf cells, respectively. Could the authors explain this discrepancy?

*Response: We thank the reviewer for the comment. The 30,230 cells (12,985 shoot cells plus 17,245 leaf cells) were the total number of single cells that were initially labeled. After cDNA libraries were generated, sequenced and filtered, 28,343 single-cell transcriptomes were obtained and subjected to further analysis in our study.*

2. Lines 112-114: Could the author conduct statistical analysis to support the significance of cell enrichment between shoot and leaf samples?

*Response: We thank the reviewer for the suggestion. We have added statistical analyses of each cell cluster to support the significance of cell enrichment between shoot and leaf samples. The results from the statistical analysis showed that clusters 3, 4, 6, 7, 8, 10, 11, 14, 15, 17, and 18 contained significantly more shoot cells than leaf cells, and clusters 0, 1, 2, 5, 9, 13, and 16 contained significantly more leaf cells than shoot cells. These results are consistent with our description in the text, except for cluster 12, which contained more cells from leaf samples, but the difference was not significant. Thus, we changed the description in the manuscript and provided significant results and figures as supplemental data (Figure S1).*

3. Lines 123-126: As written, this reviewer understands that all these marker genes are Chinese cabbage genes. However, later in the manuscript, the authors mostly cite references associated with Arabidopsis maize, peanut genes, etc. The use of orthologous (or homologous) genes should be explicitly mentioned. This reviewer was surprised that the use of this strategy between evolutionary distant plant species (e.g., Chinese cabbage and maize) could support the functional annotation of the Chinese cabbage cells.

*Response: We thank the reviewer for the comment. Yes, the marker genes used in this study are Chinese cabbage genes, which are orthologous or homologous to published marker genes in other species, including Arabidopsis, maize, and peanut.*

*During the analysis, we tested a large number of Chinese cabbage genes that are orthologous or homologous to published marker genes in other species. We found that genes with conserved gene functions were potential marker genes in Chinese cabbage, such as the chloroplast-related gene BUNDLE SHEATH DEFECTIVE 2 (BSD2), which was defined as a mesophyll cell marker gene in both Arabidopsis and maize. Therefore, some marker genes also refer to other plant species, such as maize. Due to the high homology between Chinese cabbage and Arabidopsis, many homologous genes of marker genes in Arabidopsis are expressed in Chinese cabbage, and we provided the corresponding relationship between Chinese cabbage genes and Arabidopsis genes in Table S5.*

4. Lines 224-226: The authors identified syntenic orthologs according to sequence similarity. This is not a correct approach to defining syntenic orthologs. This should be done through the analysis of conservation of chromosomal segments based on their function, order, and direction on the chromosomes.

*Response: We thank the reviewer for the comment. We agree that the identification of syntenic orthologs should be conducted through the analysis of conservation of chromosomal segments based on their function, order, and direction on the chromosomes. We defined the syntenic orthologs using the method reported by Cheng (2012), who determined syntenic genes based not only on their sequence similarity but also on the support of homologous flanking genes. We apologize for the inaccurate description, and we have revised this sentence and added a reference; see line 221-224.*

*The sentence “By determining the syntenic orthologs according to their sequence similarity between Arabidopsis and Brassica, we identified a total of 9,922 one-to-one copies…” was changed to “The syntenic orthologs between Arabidopsis and Brassica were identified using the method described by Cheng et al. (2012), and 9,922 one-to-one copies and 2,083 groups one-to-three copies of Brassica genes were identified in A03.”*

*【Cheng, F., Wu, J., Fang, L., & Wang, X. (2012). Syntenic gene analysis between Brassica rapa and other Brassicaceae species. Frontiers in plant science, 3, 198.】*

5. Lines 288-290: (1) Could the authors provide control to support that the decrease in UMI number is a response to heat stress treatment and not to the fitness of the protoplast, an indirect consequence of the stress? For instance, could the author analyze the activity of reference genes that should not be differentially expressed between control and HS treatment? Such analysis is important to conduct to support the conclusion related to the DEG analysis in response to heat stress, and the identification of cell-type-specific heat stress marker genes. (2) For instance, it was unexpected to find so many HSP genes down-regulated in response to heat stress (see lines 330-331). (3) This reviewer also suggests the use of a specifically designed bioinformatics package to identify DEGs from single-cell RNA-seq datasets (e.g. DESingle; https://bioconductor.org/packages/release/bioc/html/DEsingle.html).

*Response: We thank the reviewer for the comments.*

*(1) We referred to other single-cell experiments using related stresses, such as high-salinity treatment and low-nitrogen treatment of rice seedlings and heat-shocked treatment of Arabidopsis roots, to design and complete the heat stress experiment (Jean-Baptiste et al., 2019; Wang et al., 2020). In addition, we strictly ensured that the control group and the treatment group were consistent, including the plant size, protoplast isolation method, and single-cell labeled concentration, to ensure the accuracy of the heat stress experiment. Shahan et al. (2021) mentioned that establishing biological replicates and increasing cell numbers derived from biological replicates improves the accuracy of identifying changes in gene expression in multiconditional scRNA-seq experiments. In our study, we established two biological replicates for the control and heat treatment groups and captured a total of 37,908 cells (17,245 cells from control samples and 20,663 cells from heat treatment samples) for the gene expression analysis. Furthermore, we assessed the reference genes in different cell types between the control and treatment groups according to the reviewer’s suggestion. The Brassica reference genes UBC10, TUA, TSB, CAC, SNF, SAND, UBC1, PP2A, and ZNF (Chandna et al., 2012; Ma et al., 2020) were not differentially expressed in different cell types between the control and HS treatment groups (Table S11).*

*【Jean-Baptiste, K., McFaline-Figueroa, J. L., Alexandre, C. M., Dorrity, M. W., Saunders, L., Bubb, K. L., ... & Cuperus, J. T. (2019). Dynamics of gene expression in single root cells of Arabidopsis thaliana. The Plant Cell, 31(5), 993-1011.】*

*【Wang, Y., Huan, Q., Chu, X., Li, K., & Qian, W. (2020). Single-cell transcriptome analyses recapitulate the cellular and developmental responses to abiotic stresses in rice. BioRxiv.】*

*【Shahan, R., Nolan, T. M., & Benfey, P. N. (2021). Single-cell analysis of cell identity in the Arabidopsis root apical meristem: insights and opportunities. Journal of Experimental Botany, 72(19), 6679-6686.】*

*【Chandna, R., Augustine, R., & Bisht, N. C. (2012). Evaluation of candidate reference genes for gene expression normalization in Brassica juncea using real time quantitative RT-PCR. PloS one, 7(5), e36918.】*

*【Ma, L., Wu, J., Qi, W., Coulter, J. A., Fang, Y., Li, X., ... & Sun, W. (2020). Screening and verification of reference genes for analysis of gene expression in winter rapeseed (Brassica rapa L.) under abiotic stress. PloS one, 15(9), e0236577.】*

*(2) We also unexpectedly found that many HSPs were downregulated. However, the two biological replicates all showed that more HSPs were downregulated after high-temperature treatment for 12 hours, and thus the results obtained from this experiment were credible. According to the literature, HSP is a molecular chaperone protein, and high temperature will induce the transient expression of most HSPs, but the continuous expression of stress-responsive genes is detrimental to plant growth and development. Under long-term stress conditions, plants self-regulate and degrade stress response-related proteins to ensure plant growth and development. In this experiment, the high-temperature treatment time was 12 hours, and the plants were not under transient stress. In the late stage of high-temperature treatment, the expression of HSPs may be downregulated.*

*(3) We thank the reviewer for the suggestion. We compared the MAST and DESingle methods: MAST is based on the hurdle model, and DEsingle is based on the zero-inflated negative binomial model. We understand that algorithmic differences exist between the two software programs, but both are essentially designed to eliminate "dropout" in single-cell data. Both methods have their own advantages and are used in single-cell analyses. Among the single-cell articles that have been published in recent years, MAST has been cited 795 times, and DEsingle has been cited 116 times. The MAST method is frequently used in previous studies. In the past year, MAST has been used in single-cell articles published in Nature, Nature Neuroscience, and Nature Communications (reference are listed below). Therefore, we chose to use MAST to analyze the single-cell RNA-seq datasets.*

*【Gegenhuber, B., Wu, M. V., Bronstein, R., & Tollkuhn, J. (2022). Gene regulation by gonadal hormone receptors underlies brain sex differences. Nature, 606(7912), 153-159.】*

*【Yang, A. C., Vest, R. T., Kern, F., Lee, D. P., Agam, M., Maat, C. A., ... & Wyss-Coray, T. (2022). A human brain vascular atlas reveals diverse mediators of Alzheimer’s risk. Nature, 603(7903), 885-892.】*

*【Kamath, T., Abdulraouf, A., Burris, S. J., Langlieb, J., Gazestani, V., Nadaf, N. M., ... & Macosko, E. Z. (2022). Single-cell genomic profiling of human dopamine neurons identifies a population that selectively degenerates in Parkinson’s disease. Nature neuroscience, 25(5), 588-595.】*

*【DeVeale, B., Liu, L., Boileau, R., Swindlehurst-Chan, J., Marsh, B., Freimer, J. W., ... & Blelloch, R. (2022). G1/S restriction point coordinates phasic gene expression and cell differentiation. Nature Communications, 13(1), 1-17.】*

6. Figure 2 is another representation of Figure 1D. This reviewer suggests the deletion of Figure 2.

*Response: We removed Figure 2 from the supplemental data, as shown in Figure S2.*

7. Overall, the resolution of the figures is very low (e.g., Figure 4 was almost impossible to read).

*Response: We apologize for the inconvenience caused by the low-resolution figures in our manuscript. We adjusted the figure quality.*

Minor comments:

1. Did the authors try to evaluate the induction of protoplastization-responsive genes to better estimate the quality of their clustering?

*Response: We thank the reviewer for the comment. In the process of analyzing the data, we calculated the proportion of protoplasting genes in each cell to mitigate the effects of protoplasting on cell clustering. This analysis was used in the Arabidopsis single-cell article (Zhang et al., 2021) but was not performed in the single-cell analyses of other plant species due to limited information on protoplastization-responsive genes. Similarly, we were unable to obtain relevant gene information in Chinese cabbage, and thus this step was not included in our analysis.*

2. Line 121: Could the authors change this sentence as suggested: "…387 to 2,021 per cluster, and GO…"?

*Response: We added “per cluster” after “387 to 2,021” as according to the reviewer’s suggestion.*

3. Line 200: Could the authors make the following change: "A03…" replaced by "The Chinese cabbage A03 line…"?

*Response: We replaced “A03” with “The Chinese cabbage A03 line” according to the reviewer’s suggestion.*

4. Line 208: Please, remove "…in different cell types."

*Response: We removed the words as the reviewer suggested.*

Reviewer #2:

The authors reported a single-cell transcriptomic analysis to describe a cell atlas of Chinese cabbage leaves. The work identified 19 cell clusters, conducting a comprehensive annotation of six major cell types using known marker genes, and other novel marker genes for leaf development. This study provided new insight into studying the subgenomic evolution mechanism at the cellular level. In addition, this study underlies the mechanism on high temperature response in leaves by using the application of scRNA-seq technology. Followings are several minor comments.

*Response: Thank you for your positive comments. We agree with your constructive and insightful suggestions，please see our detailed responses to your comments below.*

Comments to author:

1. In material and method, the authors should describe more information on the analysis of gene expression differentiation among the subgenomes on single cell level. Particularly, it needs to know the criteria parameters used during the analysis.

*Response: We thank the reviewer for the comment. We modified the “Data analysis” parts in the Materials and Methods section to describe the defined differentially expressed genes among the subgenomes at the single-cell level.*

2. Could the author compare the different expression patterns of multiple gene copies in different cell types with other studies? Maybe compared to other species or tissue-level transcriptome results in discussion.

*Response: Thank you for the suggestion. We did not find articles comparing differences in subgenome expression at the cellular level, but subgenomes have dominant expression patterns at the tissue level. In two Brassica rapa accessions (Chiifu subspecies pekinensis and L58 subspecies parachinensis), genes in the less fractioned subgenome (LF) were dominantly expressed over the genes in more fractioned subgenomes (MFs: MF1 and MF2), while the genes in MF1 were slightly dominantly expressed over the genes in MF2 in different organisms, including leaf, stem and root (Cheng et al., 2012). In addition, subgenome-dominant expression is also present among the subgenomes in other plant species. In maize, a species underwent whole-genome duplication, and expression patterns of the 1,750 best-confidence pairs of maize homeologs in eight organ systems (shoots, roots, leaf tip, leaf base, bundle sheath, mesophyll, SAM and developing ear) also present dominant expression (Schnable et al., 2011). In the Discussion, we added a description of the relevant content. See lines 381-386.*

*【Cheng, F., Wu, J., Fang, L., Sun, S., Liu, B., Lin, K., ... & Wang, X. (2012). Biased gene fractionation and dominant gene expression among the subgenomes of Brassica rapa. PloS one, 7(5), e36442.】*

*【Schnable, J. C., Springer, N. M., & Freeling, M. (2011). Differentiation of the maize subgenomes by genome dominance and both ancient and ongoing gene loss. Proceedings of the National Academy of Sciences, 108(10), 4069-4074.】*

3. In discussion (L407-411), the authors described the heat responses of different cell types in Arabidopsis roots. It is possible to find the same heat stress response patterns between roots and leaves on cellular level?

*Response: Thank you for the comment. Jean-Baptiste et al. (2019) profiled the responses of 2,085 root cells (1,076 cells in the control group and 1,009 cells in the heat shock group) from 8-d-old Arabidopsis seedlings subjected to standard heat stress (45 min, 38°C). The authors identified 2,627 genes that were up- or downregulated between control and treated groups and showed differences in expression among root cell types in Arabidopsis. In our study, the genes also showed differences in expression among leaf cell types in Chinese cabbage. Because of differences in genomic information, tissue, and heat stress treatment conditions, a comparison of gene expression is difficult. Notably, we found the same functional enrichment of differentially expressed genes in Chinese cabbage leaf cells and Arabidopsis root cells. For example, genes that were differentially expressed in Chinese cabbage MC cells were enriched for ribosome-associated genes, which were also differentially expressed in Arabidopsis root hair cells upon heat shock. In summary, we have added relevant descriptions to the discussion. See lines 416-417.*

*【Jean-Baptiste, K., McFaline-Figueroa, J. L., Alexandre, C. M., Dorrity, M. W., Saunders, L., Bubb, K. L., ... & Cuperus, J. T. (2019). Dynamics of gene expression in single root cells of Arabidopsis thaliana. The Plant Cell, 31(5), 993-1011.】*

4. In Figure 4, genes names need to be italicized.

*Response: We modified the format of gene names to italics in Figure 4.*

5. In L297, the number of down-regulated genes in VC is 1,077, but in Figure 4c is 934, please check it.

*Response: We apologize for the mistake. The number of downregulated genes in VC was 934. We changed the number in the text. See line 294.*

6. Please unify the writing format of subgenome names in the whole text and figures, "LF1" or "LF"?

*Response: We carefully checked the manuscript and unified the least fractionated subgenome name to “LF” in the text and figures.*

7. The English writing of the article needs to be improved.

*Response: According to the reviewer’s suggestion, we asked a professional language editor at American Journal Experts (AJE) to help us modify the writing and typos in this manuscript.*

**Second round of review**

**Reviewer 1**

Review of the revised version of the manuscript entitled “Single-cell transcriptome reveals dominant subgenome expression and the response to heat stress in Chinese cabbage” by Sun et al and submitted for publication to Genome Biology.

I want to thank the reviewer for sharing their answers to my comments and for drastically enhancing the quality of the figures. However, this reviewer was not able to find changes in the manuscript reflecting some of the authors’ answers to my comment. Here are the specific points that require changes in the manuscript.

1- The authors did not clarify in the “Results” or “Materials and Methods” section of the manuscript the outcome of the selection of the most valuable single-cell transcriptomic datasets.

2- This reviewer did not notice a change in the inclusion of statistical analyses in Figure S1. Besides, cluster 12 is not mentioned in the manuscript as enriched in leaf cells.

3- To be consistent, could the authors also provide the orthologous relationships between Chinese cabbage genes and maize, and peanut genes as they did with the Arabidopsis genes?

**Reviewer 2**

The authors addressed the questions raised from last review. I have no other new comments, but ask the authors to improve the English writing.

**Authors Response**

**Point-by-point responses to the reviewers’ comments:**

Reviewer #1:

Review of the revised version of the manuscript entitled “Single-cell transcriptome reveals dominant subgenome expression and the response to heat stress in Chinese cabbage” by Sun et al and submitted for publication to Genome Biology. I want to thank the reviewer for sharing their answers to my comments and for drastically enhancing the quality of the figures. However, this reviewer was not able to find changes in the manuscript reflecting some of the authors’ answers to my comment. Here are the specific points that require changes in the manuscript.

*Response: Thank you for providing constructive and insightful comments. We apologize for the unclear changes to our manuscript. We have responded to your comments below and indicated the changes in the text with highlighting.*

1. The authors did not clarify in the “Results” or “Materials and Methods” section of the manuscript the outcome of the selection of the most valuable single-cell transcriptomic datasets.

*Response: We thank the reviewer for the comment. We apologize for the unclear changes to the “Results” and “Materials and Methods” sections of our manuscript. We added more information to describe the single-cell transcriptomic dataset filtering and validation to show how the valuable single-cell transcriptomic datasets were selected. Here, we list the corresponding content and the line numbers:*

*Lines 289-291: reference gene expression results*

*Lines 498-500: cell counting and quality control*

*Lines 527-536: sequencing quality analysis and low-quality cell filtration*

*Lines 541-544: batch effect correction*

*Lines 545-551: cell clustering*

*Lines 571-573: reference gene expression analysis*

2. This reviewer did not notice a change in the inclusion of statistical analyses in Figure S1. Besides, cluster 12 is not mentioned in the manuscript as enriched in leaf cells.

*Response: We thank the reviewer for the comment. We apologize for the unclear changes to the statistical analyses in the text and uploaded the incorrect Figure S1. We have now uploaded the new Figure S1, which includes bar charts with asterisks indicating significant differences in Figure S1d.*

*Figure S1. (d) Statistical analysis of cell enrichment between shoot and leaf samples in each cluster. Symbols indicate statistical significance from vehicle with p value thresholds of ≤0.05 (*), ≤0.01 (**) and ≤0.001 (***).*

*In addition, we added a description of the statistical analysis for these cell clusters and the method for drawing bar charts in the “Materials and Methods”. See lines 544-546.*

*Description: “The number of cells from shoot and leaf samples was counted in each cluster, and the cell enrichment between shoot and leaf samples was statistically analyzed by Fisher’s test. In addition, the total number of cells in each sample was normalized to 10,000 cells and then used to draw the bar charts for each cell cluster.”*

3. To be consistent, could the authors also provide the orthologous relationships between Chinese cabbage genes and maize, and peanut genes as they did with the Arabidopsis genes?

*Response: We thank the reviewer for this comment. As the reviewer suggested, we provided the orthologous relationships between Chinese cabbage genes (A03) and maize genes (B73) and those between Chinese cabbage genes (A03) and peanut genes (GCF_003086295) in Table S5.*

*The reference genomes of maize and peanut are linked below:*

*Maize: https://www.ncbi.nlm.nih.gov/genome/?term=Zea+mays*

*Peanut: https://peanutbase.org*

Reviewer #2:

The authors addressed the questions raised from last review. I have no other new comments, but ask the authors to improve the English writing.

*Response: Thank you for your positive comment. We are glad that the last rebuttal letter and the revised manuscript met with your approval. In addition, according to the reviewer’s suggestion, we asked a professional language editor at American Journal Experts (AJE) to help us improve the English writing of the manuscript.*

**Third round of review**

**Reviewer 1**

The authors addressed my questions. I have no other new comments.
